# Hydrophobic deep eutectic solvents as sustainable media for ultrasound-assisted extraction of triterpenoids: a mechanistic and experimental study on *Salvia officinalis* L.

**DOI:** 10.1039/d6ra03456j

**Published:** 2026-07-03

**Authors:** Nhan Trong Le, The-Huan Tran, Thi Thi Thi Nguyen, Thao Thi Do, Hoai Thi Nguyen

**Affiliations:** a Faculty of Pharmacy, Hue University of Medicine and Pharmacy, Hue University Hue City Vietnam nthoai@hueuni.edu.vn; b Institute of Biology, Vietnam Academy of Science and Technology Hanoi Vietnam

## Abstract

This work demonstrates that hydrophobic deep eutectic solvents (HDESs) function as environmentally benign and high-performing media for extracting triterpenoids from *Salvia officinalis* L. under ultrasound conditions. Among screened HDESs, menthol–lactic acid (Men–LA) and menthol–camphor (Men–CP) exhibited the highest extraction efficiencies. Response surface method optimization yielded triterpenoid levels of 77.27 mg g^−1^ for Men–LA and 72.76 mg g^−1^ for Men–CP, representing increases of about two to six times compared with traditional organic solvents. Kinetic modeling showed that extraction followed a second-order model, with activation energies indicating a solubilization-controlled mechanism for Men–LA and a mixed diffusion–solubilization mechanism for Men–CP. Computational analyses (ESP and IRI) revealed that strong hydrogen bonding and van der Waals interactions between solvent components and triterpenoid molecules stabilized the solute–solvent complexes, resulting in lower binding energies than ethanol. These interactions mainly involved the carboxyl/hydroxyl groups of oleanolic and ursolic acids as primary polar sites, which engaged in hydrogen bonding with polar sites of the solvent components, while van der Waals contacts were mainly associated with the pentacyclic triterpenoid cores and the hydrophobic moieties of the solvents. Coupling Men–LA with D-101 macroporous resins enabled high triterpenoid recovery (97.11%), elevated triterpenoid content (71.89%), and stable solvent recyclability (>95%), while Men–CP combined with HP-20 also performed efficiently, achieving 94.15% recovery, 68.86% content, and recyclability above 95%. The triterpenoid-rich extracts exhibited strong antioxidant and α-glucosidase inhibitory activities, surpassing those obtained with ethanol, while displaying distinct cytotoxic profiles across different cancer cell lines. Molecular docking confirmed that oleanolic acid and ursolic acid bind stably to the α-glucosidase active site, supporting their strong inhibitory effects. Overall, this integrated experimental and computational investigation highlights HDESs as a mechanistically informed and recyclable green platform for the sustainable extraction of lipophilic bioactives such as triterpenoids.

## Abstract 2

Hydrophobic deep eutectic solvents enabled high-yield extraction of Sage triterpenoids with efficient resin-based recovery, solvent reuse, and enhanced bioactivity.

## Graphical abstract

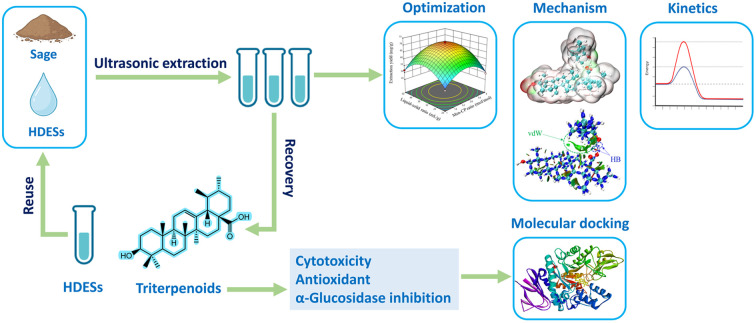

## 1. Introduction

Triterpenoids are a major class of secondary plant metabolites composed of thirty carbon atoms, biosynthetically assembled from six isoprene units. Their remarkable structural diversity underlies their wide distribution throughout the plant kingdom and accounts for their broad range of biological functions.^1^ Extensive pharmacological studies have demonstrated that triterpenoids possess diverse biological activities, including anticancer, antidiabetic, antioxidant, anti-inflammatory, antimicrobial, hepatoprotective, and cardioprotective effects.^2^ Despite their significant pharmacological potential, the extraction and isolation of triterpenoids from plant matrices remain technically challenging due to their low polarity, poor aqueous solubility, and strong association with cellular components,^3^ which collectively hinder efficient diffusion and solubilization during extraction.

Conventional extraction techniques typically utilize organic solvents including methanol, ethanol, ethyl acetate, hexane, and chloroform; however, these systems are often associated with low selectivity, elevated toxicity, and poor environmental sustainability.^4^ These drawbacks have raised increasing concerns regarding both human safety and ecological impact. Consequently, the search for greener and more sustainable alternatives to conventional solvents has become a central focus in modern natural product chemistry and pharmaceutical processing.^5^ Recent advances have led to the development of several classes of environmentally benign solvent systems, including ionic liquids (ILs),^6^ deep eutectic solvents (DESs),^7^ bio-based solvents,^8^ and surfactant-assisted extraction media.^9^ Among these, DESs have emerged as particularly promising candidates due to their tunable polarity, negligible volatility, and excellent biocompatibility, offering new opportunities for the efficient and sustainable extraction.

DESs represent an emerging class of green solvents formed through hydrogen bond interactions between a hydrogen bond donor (HBD) and a hydrogen bond acceptor (HBA).^10^ The combination of these components leads to a significant depression of the melting point compared with their individual constituents, resulting in a stable liquid phase at room temperature.^11^ Because of their low volatility, negligible flammability, and adjustable polarity,^12^ DESs have gained growing attention as effective media for isolating bioactive constituents from natural materials.^5^ However, most conventional DESs are highly hydrophilic, which restricts their ability to dissolve nonpolar compounds such as triterpenoids.^13^ To overcome this limitation, hydrophobic deep eutectic solvents (HDESs) have emerged from the combination of nonpolar or weakly polar components, typically long-chain fatty acids, terpenoids, or alcohols, to form low-polarity eutectic mixtures with enhanced affinity for hydrophobic molecules. These HDESs can establish strong van der Waals and hydrogen bonding interactions with nonpolar solutes, thereby improving solvation and mass transfer.^14,15^ Although HDESs have been investigated in several studies for extracting triterpenoids,^16–20^ most have focused on evaluating solvent performance or optimizing extraction parameters, whereas comprehensive mechanistic investigations integrating experimental and theoretical approaches remain scarce.

**Fig. 1:**
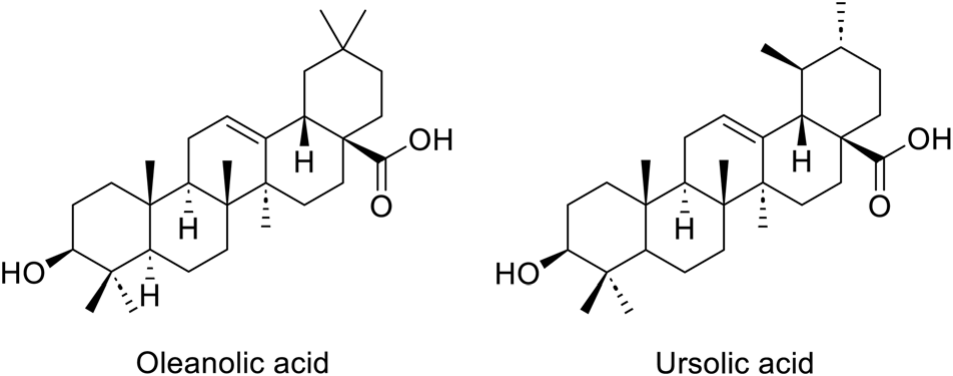
Chemical structure of oleanolic acid and ursolic acid.

*Salvia officinalis* L. (Sage) is a widely used aromatic and medicinal plant belonging to the Lamiaceae family, long recognized for its antioxidant, anticancer, antidiabetic, antibacterial, and anti-inflammatory activities.^21^ Its pharmacological potential stems from a rich chemical composition that includes phenolic acids, flavonoids, and, most notably, triterpenoids such as oleanolic acid and ursolic acid (Fig. 1),^22–24^ which are therefore selected as biomarkers for quantifying the triterpenoid fraction in this plant. While hydrophilic DESs have been widely applied to obtain phenolic components from Sage,^25–27^ comprehensive investigations addressing how green solvents can isolate triterpenoids from this species remain absent from the literature. Given the nonpolar triterpenoids, designing HDESs capable of enhancing their solubility and diffusion from the plant matrix is both scientifically important and technologically meaningful. Addressing this knowledge gap is expected to not only improve the efficiency of triterpenoid extraction from Sage but also expand the potential applications of HDES-based green solvent systems in natural triterpenoid processing.

Accordingly, this work develops and evaluates HDESs as eco-friendly media for ultrasonic triterpenoid extraction, using Sage as a model system. A series of naturally derived HDESs were formulated and screened to identify the most efficient solvent systems, followed by optimization of extraction conditions using response surface methodology. The extraction kinetics and the molecular-level interactions between triterpenoids and solvent components were systematically examined through both experimental and computational approaches, providing insights into the mechanism of HDESs-assisted extraction. Furthermore, triterpenoid recovery and solvent recyclability were evaluated to confirm the feasibility and sustainability of the proposed process. This integrated investigation bridges the gap between empirical optimization and molecular understanding, establishing HDESs as a practical and mechanistically informed platform for the green extraction of lipophilic bioactive compounds.

## 2. Materials and methods

### 2.1. Materials and chemicals

Aboveground parts of Sage (*Salvia officinalis* L.) were purchased from a local herbal pharmacy in Hue City, Vietnam, in August 2024. The plant material was cleaned, air-dried, and ground into a fine powder, which was then sieved through a 0.71 mm mesh to ensure uniform particle size. The powdered sample was stored in airtight, light-protected containers until further use.

Standard compounds, including ursolic acid (UA) and oleanolic acid (OA), were obtained from Macklin Inc. (Guangdong, China). Chemical reagents for preparing HDESs consisted of menthol, lactic acid, propionic acid, acetic acid, valeric acid, pyruvic acid, butyric acid, nonanoic acid, octanoic acid, capric acid, camphor, thymol, and lauric acid. Conventional solvents such as methanol, ethanol, ethyl acetate, *n*-hexane, chloroform, sodium hydroxide, and hydrochloric acid were purchased from Macklin and Xilong (Guangdong, China). Detailed information regarding the chemicals and reagents used in this study, including CAS numbers, purities, and commercial suppliers, is provided in Table S1.

A range of macroporous resins (MRs), including HP-20, LSA-40, HPD-300, ADS-7, D-101, DM-301, XAD-8, AB-8, and HPD-400, were purchased from Hecheng New Material Technology Co., Ltd (Zhengzhou, China) and Supelco™ Analytical (Pennsylvania, USA).

Human cell lines, including MCF-7 (breast carcinoma), HepG2 (hepatocarcinoma), A549 and SK-LU-1 (lung carcinoma), AGS (gastric carcinoma), HT-29 (colorectal carcinoma), SW-480 (colon carcinoma), HeLa (cervical carcinoma), and HEK-293A (embryonic kidney), were provided by the Long Island University in the USA and the University of Milan in Italy. All reagents used in the biological assays were obtained from commercial suppliers, as detailed in Table S1.

The principal instruments included a magnetic stirrer with heating (Labnet, USA) was employed for HDESs preparation, an ultrasonic bath (S 100H, 37 kHz, 150 W, Elma, Germany) for extraction, a centrifuge (Zhengji, China) for separating the solid residues from the extract, a rotary evaporator (R-300, Buchi, Switzerland) for concentrating the filtrate, a reverse-phase HPLC system (Agilent 1260 Infinity II, USA) for quantitative analysis, and a Bruker Avance NEO 600 MHz spectrometer (Bruker, USA) for ^1^H NMR measurements.

### 2.2. Solvent preparation and selection

The HDES formulations were obtained by combining the chosen components in predetermined molar proportions, as listed in Table 1. The prepared components were warmed to temperatures under 80 °C and kept in constant agitation using a laboratory stirring device for 2 to 4 hours until a uniform and stable liquid formed. The prepared HDESs were then cooled to room temperature and allowed to equilibrate for 24 h before use. All solvents were stored in tightly sealed bottles inside a desiccator to prevent moisture absorption.

**Table 1:** Composition of HDESs used for solvent screening in the extraction of triterpenoids from Sage

Type	Extraction solvents	Abbreviation	Molar ratio (mol : mol)
Menthol-based HDESs	Menthol–acetic acid	Men–AA	1 : 1
	Menthol–lactic acid	Men–LA	1 : 1
	Menthol–propionic acid	Men–PrA	1 : 1
	Menthol–pyruvic acid	Men–PyA	1 : 1
	Menthol–butyric acid	Men–BA	1 : 1
	Menthol–octanoic acid	Men–OA	1 : 1
	Menthol–nonanoic acid	Men–NA	1 : 1
	Menthol–capric acid	Men–CA	1 : 1
	Menthol–camphor	Men–CP	1 : 1
	Menthol–thymol	Men–TM	1 : 1
	Menthol–lauric acid	Men–LaA	2 : 1
Lauric acid–based HDESs	Lauric acid–octanoic acid	LaA–OA	1 : 3
	Lauric acid–nonanoic acid	LaA–NA	1 : 3
	Lauric acid–capric acid	LaA–CA	1 : 3
Traditional organic	Ethanol	EtOH	—
	Methanol	MeOH	—
	Ethyl acetate	EtOAc	—
	*n*-Hexane	*n*-Hexane	—
	Chloroform	Chloroform	—

For the solvent screening step, identical extraction conditions were applied to all HDES formulations, consisting of a liquid to solid ratio of 20 mL per gram, a 30 minute extraction period, and a working temperature of 50 °C. Following extraction, the samples were centrifuged at 4000 rpm for five minutes to obtain the clarified supernatant. The triterpenoid concentration in the extract was determined using HPLC analysis.

### 2.3. Optimization of triterpenoid extraction

Response Surface Methodology (RSM) coupled with a Box–Behnken design (BBD) was applied to optimize the extraction of triterpenoids from Sage. Four factors, including the HBA to HBD molar proportion, the liquid to solid ratio, the extraction duration, and the extraction temperature, were treated as independent variables. The combined yield of oleanolic acid and ursolic acid (T-OU) served as the response variable. The coded factors and their corresponding experimental levels applied in the optimization procedure are provided in Tables S2 and S3.

### 2.4. Kinetic investigation of triterpenoid extraction

The extraction kinetics of triterpenoids from Sage using HDESs were interpreted using a second-order model to describe the mass transfer process. The relationship between extraction time (*t*) and the amount extracted (*C*_*t*_) is expressed as:
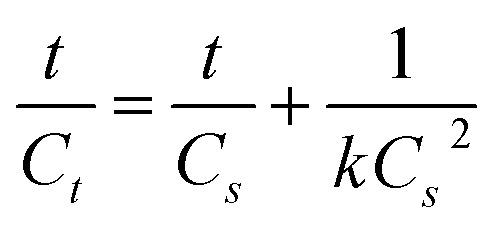
where *C*_t_ and *C*_s_ (mg g^−1^) are the triterpenoid yields at time *t* and equilibrium, respectively; and *k* (g mg^−1^.min^−1^) is the rate constant. The initial extraction rate (*h*) was calculated as:*h* = *kC*_s_^2^

Temperature dependence of *k* followed the Arrhenius equation,
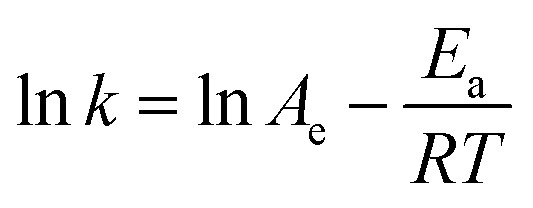
where *A*_e_ (mg g^−1^.min^−1^) is the frequency factor, *E*_a_ (kJ mol^−1^) is the activation energy, *R* is the gas constant, and *T* (K) is the absolute temperature.

The kinetic parameters were estimated by nonlinear regression and Arrhenius analysis to elucidate the rate and mechanism of triterpenoid release from the plant matrix.

### 2.5. Quantification of OA and UA by HPLC

The concentrations of OA and UA in the samples were determined using a reverse-phase HPLC system.^28^ Separation was achieved on an Eclipse XDB-C18 column (4.6 × 250 mm, 5.0 µm; Agilent Technologies). The mobile phase was composed of 90 percent methanol and a 10 percent aqueous solution containing 0.1 percent trifluoroacetic acid. Chromatographic separation was performed using a flow rate of 0.6 mL per minute, with the detector set at 205 nm. Representative chromatograms of the samples and standards are displayed in Fig. S1.

### 2.6. Triterpenoid recovery and reuse of HDESs and MRs

A solid–liquid extraction (SLE) method combined with MRs was implemented to recover triterpenoids from HDESs extracts of Sage. Each MRs (10 g) was pretreated with ethanol, dilute sodium hydroxide, and dilute hydrochloric acid, then washed with distilled water to neutral pH and packed into glass columns measuring 10 mm in diameter and 25 cm in length. A 2 mL aliquot of the optimized HDESs extract was applied, and the column was rinsed with 60% ethanol to remove residual solvents. Triterpenoids were then eluted with absolute ethanol, and the eluates were evaporated to obtain triterpenoid-rich fractions.

For reuse, the ethanol fractions containing HDESs residues were concentrated to regenerate the solvents. The exhausted MRs were reactivated by sequential washing with dilute sodium hydroxide, dilute hydrochloric acid, and distilled water. Both HDESs and MRs after regeneration were employed in three consecutive extraction–recovery cycles. The recyclability of the system was evaluated by monitoring extraction yield, triterpenoid recovery, triterpenoid content, and the recovery efficiency of HDESs.

### 2.7. Biological activities

#### 2.7.1. Cytotoxicity assay

Cytotoxic effects were evaluated using the sulforhodamine B (SRB) method.^29^ Cells were maintained in DMEM containing 10% fetal bovine serum, 2 mM l-glutamine, and 1.5 g L^−1^ sodium bicarbonate at 37 °C in a 5% CO_2_ atmosphere. For the assay, 5700 cells per well were seeded in 96-well plates, allowed to attach overnight, and then treated with sample solutions for 72 h. After incubation, cells were fixed with cold 20% trichloroacetic acid, stained with SRB, washed with 1% acetic acid, and solubilized in 10 mM Tris base. The negative and positive controls were 10% DMSO and ellipticine, respectively. Readings were taken at a wavelength of 515 nm. The inhibition rate (I%) was computed based on the equation: *I*% = [1 − (OD_t_ − OD_0_)/(OD_c_ − OD_0_)] × 100, where OD_t_, OD_0_, and OD_c_ denote the absorbance values of treated cells after 72 h, the initial reading, and the untreated control, respectively.

#### 2.7.2. Antioxidant assay

The antioxidant capacity of the extracts was evaluated through their DPPH radical scavenging potential.^30^ A methanolic DPPH solution (0.25 µM) was freshly prepared and mixed with an equal volume of each sample. After vortexing, the reaction mixtures were incubated for 30 min at ambient temperature in the dark. The decrease in absorbance was monitored with a UV-vis spectrophotometer at 517 nm. Methanol served as the blank, while l-ascorbic acid was used as a reference antioxidant. The scavenging percentage (*I*%) was obtained using the equation *I*% = [(*A*_0_ − *A*_s_)/*A*_0_] × 100, where *A*_0_ and *A*_s_ correspond to the absorbance of the control and the sample, respectively.

#### 2.7.3. α-Glucosidase inhibitory assay

The *in vitro* α-glucosidase inhibition assay was performed using a procedure adapted from Li *et al.*^31^ Sample solutions at various concentrations were combined with α-glucosidase (0.2 U mL^−1^) that had been prepared in 100 mM phosphate buffer at pH 6.8, followed by incubation at 37 °C for 10 min. The reaction was initiated by adding 50 µL of *p*-nitrophenyl-α-d-glucopyranoside, maintained for 30 min, and stopped with 100 µL of 0.1 M Na_2_CO_3_. The optical density at 410 nm was determined, with acarbose employed as the positive control. Enzyme inhibition was calculated as *I*% = (1 − *A*_sample_/*A*_control_) × 100, where *A*_sample_ and *A*_control_ correspond to the absorbance values in the presence and absence of the test sample.

### 2.8. Computational methods

#### 2.8.1. Quantum chemical calculations

Computational modeling was employed to clarify the molecular interactions between triterpenoids and solvent constituents in HDESs. All calculations were conducted using ORCA 6.1.0.^32–35^ The molecular structures of OA, UA, Men, LA, CP, and EtOH were constructed and energy-minimized in Avogadro^36^ with the MMFF94 force field. Optimized geometries were obtained through gas-phase DFT calculations at the B3LYP-D3(BJ)/def2-SVP level, applying RIJCOSX acceleration and TightSCF convergence. Multiwfn 3.8 (ref. 37 and 38) was used to analyze electrostatic potential (ESP) and interaction region indicator (IRI)^39^ features to visualize intermolecular solute–solvent interaction regions, while graphical outputs were prepared in VMD 1.9.4.^40^ Binding energies (Δ*E*_binding_) were derived from:Δ*E*_binding_ = *E*_complex_ − (*E*_solute_ + *E*_solvent_)

More negative Δ*E*_binding_ values indicate stronger and more favorable solute–solvent associations.

#### 2.8.2. Molecular docking

Molecular docking simulations were carried out using AutoDock Vina 1.1.2 (ref. 41) to investigate the interactions between OA and UA with α-glucosidase (PDB: 5ZCC).^42^ The enzyme structure was retrieved from the Protein Data Bank and prepared in AutoDock Tools 1.5.7 (ref. 43) by removing water molecules and non-essential ligands, then adding Kollman charges and polar hydrogens. The ligand structures were drawn in ChemDraw 16.0, converted to 3D forms, and energy-minimized using Open Babel 3.1.1.^44^ Docking was performed with an exhaustiveness of 64, focusing on the catalytic pocket defined by the co-crystallized inhibitor. Binding poses were ranked according to their binding affinities (Δ*G*_binding_, kcal mol^−1^), and the best conformations were analyzed in BIOVIA Discovery Studio 24.1 to identify key hydrogen bonds and hydrophobic interactions contributing to enzyme inhibition.

### 2.9. Statistical analysis

All experiments were performed in triplicate, and results were expressed as mean ± standard deviation. Statistical evaluation was performed through one-way ANOVA, and pairwise differences were determined using the least significant difference (LSD) test at a significance cutoff of *p* < 0.05. Optimization of extraction variables was carried out using the Design-Expert software (v13.0, Stat-Ease Inc., Minneapolis, USA) employing RSM approach.

## 3. Results and discussion

### 3.1. Screening of HDESs

The choice of solvents is pivotal in governing both extraction efficiency and selectivity toward triterpenoids. HDESs based on menthol and lauric acid were employed as green solvents for preliminary screening. This selection is justified by the relatively low polarity of HDESs, which facilitates the dissolution and extraction of weakly polar triterpenoids such as OA and UA, resulting in superior extraction efficiency compared with traditional organic solvents. For comparison, ethanol (EtOH), methanol (MeOH), ethyl acetate (EtOAc), *n*-hexane, and chloroform were employed as control conventional solvents.

The main aim of this screening step was to determine which solvents were suitable for extracting triterpenoids from Sage. To achieve this, the extraction yields of the two major triterpenoids, OA and UA, as well as their combined extraction yield (T-OU), were determined. For consistency and comparability across different solvent systems, the extraction parameters were fixed at a solvent-to-material ratio of 20 : 1 (mL g^−1^), an extraction temperature of 50 °C, and an extraction time of 30 min.

**Fig. 2:**
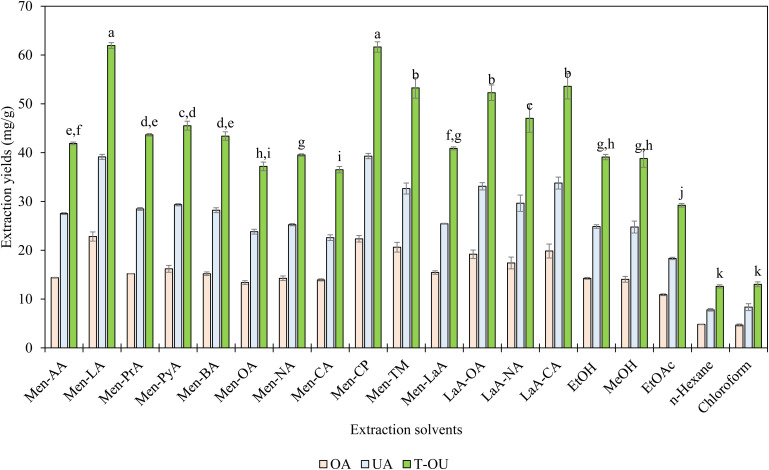
Extraction yields of triterpenoids from Sage using HDES solvents. Columns marked with different letters represent groups that differ significantly in T-OU yields at *p* < 0.05.

The levels of OA, UA and T-OU extracted using different HDESs are illustrated in Fig. 2, while the corresponding extraction values are detailed in Table S4. For menthol-based HDESs, the yields ranged from 13.37 to 22.81 mg g^−1^ for OA, 22.58 to 39.30 mg g^−1^ for UA, and 36.51 to 61.94 mg g^−1^ for T-OU. Lauric acid-based HDESs exhibited extraction yields ranging from 17.40 to 19.84 mg g^−1^ for OA, 29.64 to 33.76 mg g^−1^ for UA, and 47.04 to 53.60 mg g^−1^ for T-OU.

Among all tested HDESs, menthol–lactic acid (Men–LA) and menthol–camphor (Men–CP) demonstrated the most efficient extraction of triterpenoids from Sage. Relative differences are detailed in Table S4. The maximum amounts of OA, UA and T-OU obtained with Men–LA were 22.81 ± 0.94 mg g^−1^, 39.13 ± 0.51 mg g^−1^ and 61.94 ± 0.59 mg g^−1^, respectively, while those obtained with Men–CP were 22.35 ± 0.66 mg g^−1^, 39.30 ± 0.55 mg g^−1^, and 61.64 ± 1.06 mg g^−1^, respectively. In comparison, the amounts extracted using traditional organic solvents (EtOH, MeOH, EtOAc, *n*-hexane, and chloroform) were considerably lower than those achieved with HDESs. Specifically, the yields of OA, UA, and T-OU ranged from 4.68 to 14.24 mg g^−1^, 7.80 to 24.86 mg g^−1^, and 12.60 to 39.10 mg g^−1^, respectively.

The extraction performance of HDESs can be interpreted through the combined effects of intermolecular interactions, solvent composition, and physicochemical properties.^45^ van der Waals interactions between the hydrophobic solvent domains and the triterpenoid skeleton promote stabilization of nonpolar regions, whereas hydrogen bonding between solvent functional groups and OA or UA enhances solubility through specific polar interactions. Accordingly, extraction efficiency depends not solely on the HBA or HBD individually but rather on their cooperative interactions within the HDESs system.^13^

This effect was evident from the different performances observed among the investigated HDESs. Although menthol and lauric acid served as the principal HBA, their extraction behavior varied markedly depending on the accompanying HBD. Only specific menthol-based systems, particularly Men–LA and Men–CP, achieved superior triterpenoid extraction, whereas other menthol combinations exhibited comparable or lower yields than lauric acid-based systems. Furthermore, HDESs formulated with short-chain acids generally showed better extraction performance than those containing longer aliphatic acids. Shorter acids provide higher polarity and stronger hydrogen-donating ability, facilitating triterpenoid solvation and release from the plant matrix. In contrast, longer carbon chains promote solvent self-association and reduce hydrogen-bonding capability, weakening solvent–solute interactions and resulting in lower extraction efficiencies.

Viscosity also influenced solvent behavior and mass transfer during extraction. Literature-reported viscosity values for the investigated HDESs ranged from approximately 7.1 to 56.7 mPa s.^13,15,46,47^ Men–LA and Men–TM exhibited relatively high viscosities of 56.7 and 53.1 mPa s, respectively, whereas Men–CP showed an intermediate viscosity of 23.0 mPa s and lauric acid-based systems such as LaA–OA and LaA–NA displayed lower viscosities of 7.1 and 8.6 mPa s, respectively.^13,47^ However, no simple linear relationship between viscosity and extraction efficiency was observed. Men–LA, despite possessing one of the highest viscosity values, produced the highest triterpenoid yield, indicating that viscosity alone could not explain solvent performance and that polarity together with intermolecular interactions remained equally important.

Among all tested systems, Men–LA and Men–CP exhibited the best extraction performance. The superior efficiency of Men–LA can be attributed to the hydroxyl group of lactic acid, which enables stronger and more extensive hydrogen bonding with OA and UA compared with propionic acid. In the case of Men–CP, the hydrogen-bond-accepting ability and rigid bicyclic structure of camphor likely promoted favorable dipolar and dispersion interactions with triterpenoid molecules. These findings demonstrate that triterpenoid extraction using HDESs depends on a delicate balance of polarity, viscosity, and intermolecular interactions, highlighting the importance of rational solvent design for sustainable triterpenoid enrichment from Sage.

### 3.2. Optimization of triterpenoid extraction process

The triterpenoid extraction from Sage using Men–LA and Men–CP HDESs was optimized through a four-factor BBD (Tables S2 and S3). The results of the 29 experimental runs are summarized in Tables S5 and S6.

Analysis of variance (ANOVA) for both HDES systems (Tables S7 and S8) demonstrated that the models were statistically significant (*p* < 0.0001), with *F*-values of 120.71 for the Men–LA and 73.97 for the Men–CP. Moreover, the lack-of-fit tests were insignificant (*p* > 0.05), confirming that the models adequately represented the experimental data. The correlation between experimental and predicted responses is depicted in Tables S9 and S10 and Fig. S2 and S3, where all *R*^2^ values exceeded 0.94, indicating excellent model reliability and predictive capability. The second-order polynomial equations describing the relationships between triterpenoid yield and the experimental variables are expressed as follows:*Y*_1_ = − 672112 − 8,00204*A* + 124292*B* + 0328627*C* + 155087*D* − 0019301*AB* + 0003785*AC* + 0185225*AD* + 0010167*BC* + 0005564*BD* − 0000273*CD* − 0219685*A*^2^ − 0,021425*B*^2^ − 0006395*C*^2^ − 0017196*D*^2^*Y*_2_ = 270293 + 1732180*A* + 218904*B* + 0087315*C* + 0018501*D* + 0068915*AB* + 0092770*AC* − 0065463*AD* + 0018366*BC* + 0002045*BD* − 0006860*CD* − 371274*A*^2^ − 0042340*B*^2^ − 0007433*C*^2^ + 0003248*D*^2^

In these equations, *Y*_1_ and *Y*_2_ (mg g^−1^) represent the extraction yields of total triterpenoids obtained with the Men–LA and Men–CP HDESs, respectively. The variables *A* (mol mol^−1^), *B* (mL g^−1^), *C* (min), and *D* (°C) correspond to the HBA/HBD molar ratio, liquid-to-solid ratio, extraction time, and extraction temperature, respectively.

**Fig. 3:**
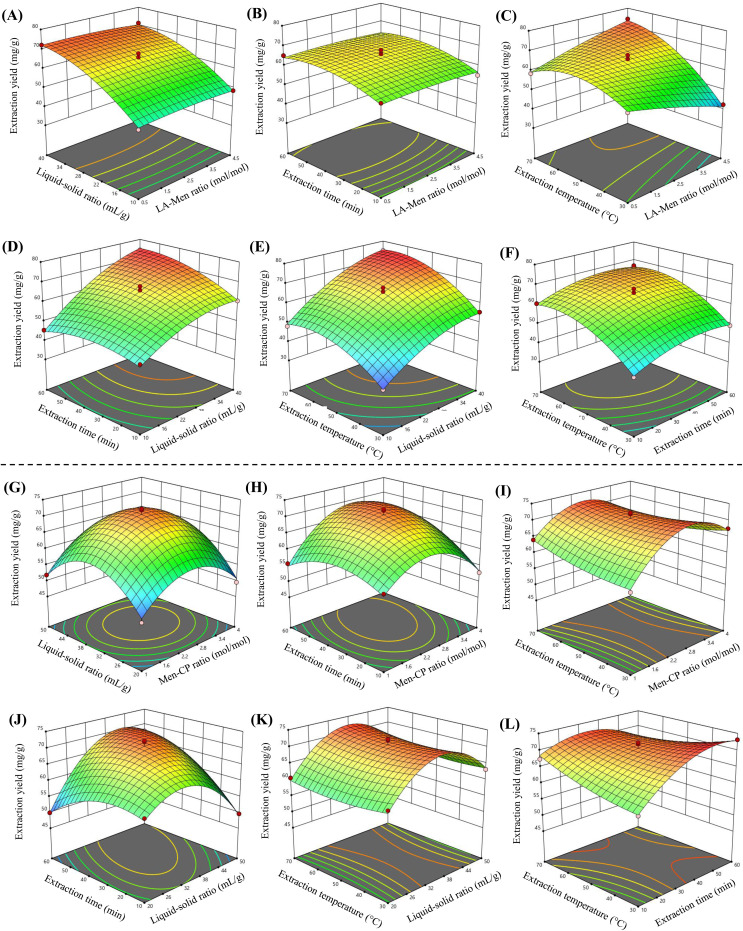
Response surface analysis of the combined effects of extraction parameters on triterpenoid yield from Sage using Men–LA (A–F) and Men–CP (G–L) HDESs.

The effects of individual extraction factors and their interactive influences on triterpenoid yield are illustrated in Fig. 3. The influence of the main extraction factors on triterpenoid yield was further evaluated for both HDES systems. The HBA/HBD molar ratio showed contrasting behaviors. In the Men–LA system, the LA-to-Men ratio exerted no significant effect (*p* = 0.3614), resulting in negligible changes in yield (Fig. 3A–C), whereas in the Men–CP system, the Men-to-CP ratio had a pronounced impact (*p* = 0.0002), with yield increasing up to approximately 2.5 mol mol^−1^ before gradually declining (Fig. 3G–I). The liquid-to-solid ratio significantly affected extraction efficiency for both HDESs (*p* < 0.0001), though their response patterns differed. In the Men–LA system, higher solvent volumes consistently enhanced yield (Fig. 3A, D and E), while in the Men–CP system, yield peaked at around 36 mL g^−1^ and decreased thereafter (Fig. 3G, J and K). This trend can be explained by the initial increase in concentration gradient that facilitates solute dissolution and diffusion, followed by a dilution effect at excessive solvent volumes, which reduces the driving force for mass transfer and lowers the effective concentration of triterpenoids in the extract.^48^ Extraction time also exhibited a strong influence (*p* < 0.0001), with yield rising rapidly during the initial stage and reaching maxima at approximately 40 min for Men–LA and 37 min for Men–CP before slightly declining (Fig. 3B, D and F for Men–LA; Fig. 3H, J and K for Men–CP). The rapid extraction observed in the early stage primarily results from the large concentration difference between the plant matrix and the HDES, which drives the diffusion of triterpenoids into the solvent phase. As equilibrium is approached, mass transfer slows, and prolonged extraction, particularly at high temperatures, can partially disrupt the hydrogen bonding network and supramolecular organization of the HDESs, increasing viscosity and reducing extraction efficiency. Extraction temperature displayed a significant influence in the Men–LA system (*p* < 0.0001) but a negligible one in the Men–CP system (*p* = 0.5334). In the former, increasing temperature enhanced triterpenoid yield up to around 65 °C, after which a slight decline was observed (Fig. 3C, E and F), whereas in the latter, yield remained nearly unchanged with temperature variation (Fig. 3I, K and L).

The simultaneous interactions among extraction parameters had a remarkable influence on triterpenoid yield when using both Men–LA and Men–CP HDESs (Fig. 3). In the Men–LA system (Fig. 3A–F), the most significant interactions were observed between the LA-to-Men ratio and extraction temperature (*p* < 0.0001), between the liquid-to-solid ratio and extraction time (*p* < 0.0001), and between the liquid-to-solid ratio and temperature (*p* = 0.0308). Concurrent increases in these variables resulted in a clear enhancement of triterpenoid yield (Fig. 3C–E), whereas other factor combinations did not show significant effects (*p* > 0.05). These findings suggest that solvent composition and extraction temperature cooperatively regulate HDESs properties and solvent penetration into the plant matrix. A higher liquid-to-solid ratio promoted triterpenoid solubilization at moderate temperatures, although this effect weakened at elevated temperatures, likely due to partial disruption of the hydrogen-bonding network governing HDESs stability. For the Men–CP system, several notable interaction effects were identified, including between the Men-to-CP ratio and liquid-to-solid ratio (*p* = 0.0224), the Men-to-CP ratio and extraction time (*p* < 0.0001), the Men-to-CP ratio and extraction temperature (*p* = 0.0058), the liquid-to-solid ratio and extraction time (*p* < 0.0001), and the extraction time and temperature (*p* < 0.0001). Most interactions exhibited an initial increase in yield followed by a decline (Fig. 3G–L), indicating a narrower optimal operating range for this system. Moderate Men-to-CP ratios combined with sufficient extraction time favored triterpenoid release through improved solvation and diffusion, whereas excessive conditions likely increased viscosity and reduced solvent accessibility within the biomass. Overall, these interaction effects demonstrate that both solvent composition and extraction conditions dynamically govern the solvating capacity and extraction efficiency of HDESs. The quadratic terms (*B*^2^, *C*^2^, and *D*^2^) were also significant, confirming the curvature of the response surface and suggesting that extreme low or high levels of each variable can lead to a decrease in triterpenoid yield, consistent with the nonlinear nature of the extraction.

For the Men–LA formulation, the model defined the best operating settings as an LA to Men proportion of 2.8 mol per mol, a solvent load of 36.4 mL per gram of plant material, together with 40 min of extraction at 65 °C. In the case of the Men–CP system, the predicted optimal region corresponded to a Men to CP molar ratio of 2.56, a solvent dosage of 38.2 mL per gram, an extraction period of 37 min, and a working temperature of 70 °C. Under these optimized conditions, the T-OU yields obtained from the Men–LA and Men–CP systems were 77.27 and 72.76 mg g^−1^, respectively.

### 3.3. Extraction mechanism

To obtain a more comprehensive understanding of triterpenoid extraction at the molecular scale, the interaction mechanisms between the target compounds and the components of HDESs were systematically investigated. The remarkably enhanced extraction performance of the two HDESs compared to conventional solvents is governed by interactions occurring between the triterpenoid framework and the solvent constituents. To elucidate this mechanism, computational analyses including ESP mapping and IRI visualization were performed to characterize the nature and strength of noncovalent interactions between triterpenoid molecules (OA and UA) and individual components of the Men–LA and Men–CP HDESs (Men, LA, and CP). Ethanol (EtOH) was used as a reference solvent for comparison, as it exhibited measurable triterpenoid extraction efficiency from Sage.

**Fig. 4:**
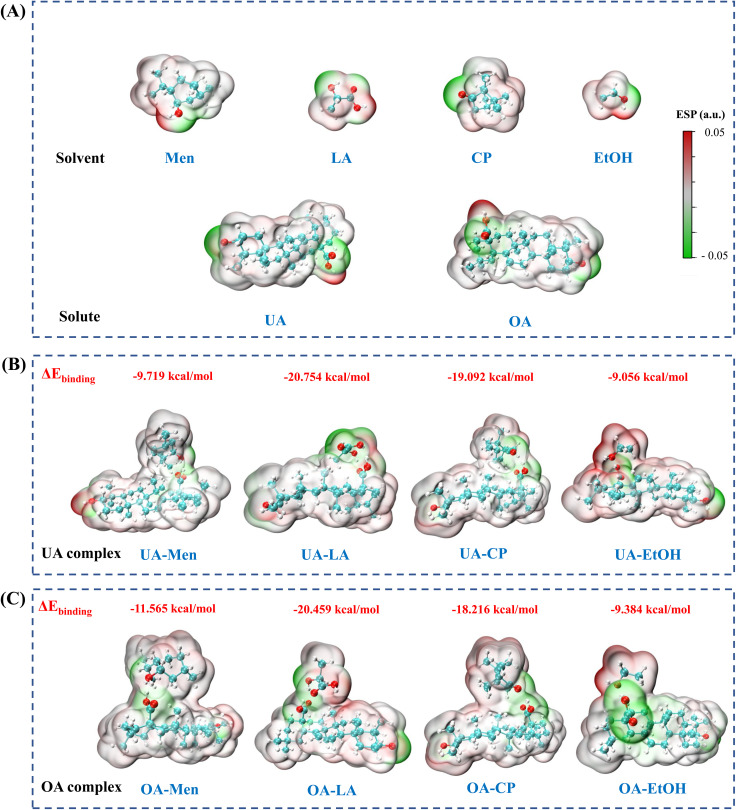
ESP maps and binding energies illustrating the interactions between triterpenoid (OA and UA) with individual components of the Men–LA and Men–CP HDESs, in comparison with the conventional solvent (EtOH). (A) ESP surfaces of individual molecules, including Men, LA, CP, EtOH, UA, and OA. (B) Interaction configurations and binding energies of UA with Men, LA, CP, and EtOH. (C) Interaction configurations and binding energies of OA with Men, LA, CP, and EtOH.

The ESP maps illustrate electron-rich regions represented in green and electron-deficient regions shown in red. The results revealed that electron-rich regions were mainly located around the oxygen atoms of the carboxylic acid and hydroxyl groups in Men, LA, EtOH, UA, and OA, as well as around the carbonyl group in CP. In contrast, electron-deficient regions were typically distributed around the labile hydrogen atoms of hydroxyl groups in Men, LA, EtOH, UA, and OA (Fig. 4A). These electron-rich and electron-deficient regions tend to interact strongly through mutual electron complementarity, indicating that the binding sites between solvent components and OA or UA are primarily concentrated within these regions (Fig. 4B and C).

The interactions between UA and the individual components of the HDESs composed of Men–LA and Men–CP were compared with ethanol. The calculated binding energies indicated that the HDES–UA complexes were significantly more stable than the EtOH–UA complex (Fig. 4B), reflecting stronger molecular interactions between the triterpenoid and the HDES components, thereby explaining the superior extraction efficiency of these systems compared with conventional organic solvents. Specifically, the binding energies of UA–Men, UA–LA, UA–CP, and UA–EtOH were −9.719, −20.754, −19.092, and −9.056 kcal mol^−1^, respectively. The markedly lower Δ*E*_binding_ values of UA–LA and UA–CP compared with UA–Men demonstrate that LA and CP are the key components responsible for forming stable polar interactions with UA.

**Fig. 5:**
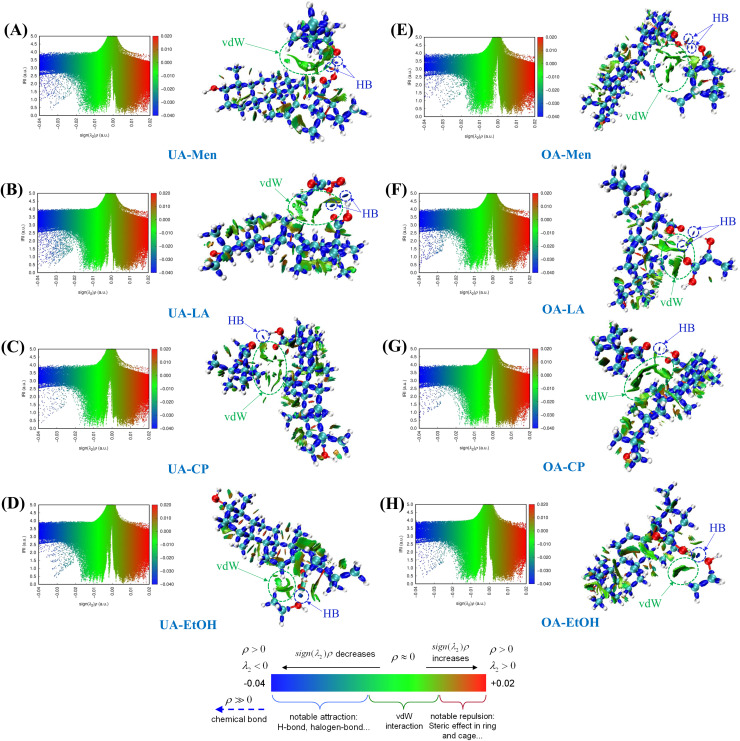
IRI scatter plots and isosurface representations illustrating the noncovalent interaction regions between UA with Men, LA, CP, and EtOH (A–D), and OA with Men, LA, CP, and EtOH (E–H). The visualizations highlight the presence of hydrogen bonding (HB) and van der Waals (vdW) interactions.

The IRI analysis (Fig. 5A–D) provides visual evidence supporting this difference. In the UA–EtOH system, only a small blue region corresponding to a single hydrogen bond between the –OH group of EtOH and the –COOH group of UA was observed, accompanied by a narrow green region representing weak van der Waals interactions, indicating overall weak interaction and limited solubility. In contrast, the UA–Men, UA–LA, and UA–CP complexes exhibited pronounced blue regions and broad green areas surrounding the pentacyclic skeleton of UA, revealing the coexistence of directional hydrogen bonds and significantly stronger van der Waals interactions than those with EtOH. Among them, LA exhibited a dominant role due to its ability to act as both a proton donor and acceptor, forming a multi-site hydrogen-bonding network between its hydroxyl and carbonyl groups and the carboxyl and hydroxyl groups of UA, while maintaining van der Waals interactions along the alkyl chain, resulting in the most stable complex. CP, although lacking a hydroxyl group, formed O–H⋯O=C hydrogen bonds *via* its polar carbonyl group and generated large hydrophobic contact areas through its bicyclic structure, which further stabilized the complex. In contrast, Men formed only one weak local hydrogen bond and displayed a smaller van der Waals interaction surface, playing an auxiliary role in creating a hydrophobic environment that enhances the solubility of UA in the solvent. In other words, Men does not act as a major binding center but rather functions as a “molecular cage” that allows LA or CP to anchor UA through hydrogen bonding.

A similar trend was observed for OA, with the binding energies of OA–LA (−20.459 kcal mol^−1^) and OA–CP (−18.216 kcal mol^−1^) being notably lower than those of OA–Men (−11.565 kcal mol^−1^) and OA–EtOH (−8.384 kcal mol^−1^) (Fig. 4C). The IRI analysis (Fig. 5E–H) further confirms the predominant role of LA and CP in forming strong noncovalent interactions with OA. In the OA–LA complex, blue regions were clearly distributed around the –COOH and –OH groups of OA, indicating a multi-point hydrogen-bonding network between the two molecules, while the extended green areas along the alkyl chain illustrate additional stabilization through van der Waals interactions. The OA–CP complex showed hydrogen-bonding regions centered around the carbonyl group of CP and the –COOH group of OA, along with dense van der Waals areas, suggesting that CP can simultaneously contribute to both polar and hydrophobic interactions. In contrast, the OA–Men complex exhibited weaker and more limited interaction regions, reflecting Men's supporting role in providing a hydrophobic microenvironment and enhancing solubility of the hydrocarbon skeleton of OA rather than participating directly in strong hydrogen bonding.

Overall, the combination of strong polar hydrogen bonds (from LA and CP) and the hydrophobic microenvironment shaped by Men enables efficient solvation and stabilization of triterpenoids in HDESs. The cooperative effect between these interactions results in lower binding energies and enhanced extraction efficiency compared with ethanol. Collectively, these computational insights demonstrate that the superior performance of Men–LA and Men–CP HDESs originates from the complementary roles of their components—LA and CP providing strong directional hydrogen bonding, while Men modulates the solvent polarity and hydrophobicity to optimize triterpenoid dissolution and mass transfer.

### 3.4. Extraction kinetics

The extraction of biomolecules from plant matrices is generally governed by solubilization and diffusion mechanisms, which depend on the physicochemical properties of the solvent and extraction conditions. To accurately describe the relationship between extraction time and yield, the second-order kinetic model was employed to determine the characteristic parameters reflecting extraction rate and mechanism. This model assumes that the extraction rate is proportional to the square of the difference between the equilibrium concentration and the concentration at a given time, representing the combined effects of diffusion and solubilization during mass transfer. Investigating the kinetics based on the second-order model allows the determination of key parameters (*k*, *h*, *C*_s_) and provides insights into how different HDESs systems influence the extraction mechanism.

The experiments were performed at three temperatures (40 °C, 50 °C, and 60 °C) and four extraction times (5, 20, 35, and 50 min). The obtained data were fitted to the second-order kinetic model, and the linear plots of *t*/*C*_*t*_*versus t* are presented in Fig. S4–S7. The obtained kinetic parameters are presented in Table 2. The findings indicated that the second-order kinetic model provided an excellent fit for the triterpenoid extraction process using HDESs, with *R*^2^ values greater than 0.98 under all tested conditions.

**Table 2:** Second-order kinetic parameters describing triterpenoid extraction from Sage using Men–LA and Men–CP HDESs^a^

	*T* (^o^C)	*C*_s_ (mg g^−1^)	*h* (mg g^−1^.min^−1^)	*k* (g mg^−1^.min^−1^)	*R*_1_^2^	*E*_a_ (kJ mol^−1^)	ln *A*_e_	*R*_2_^2^
Men-LA	40	60.24	17.79	0.0049	0.9851	60.84	18.029	0.9933
	50	62.11	35.34	0.0092	0.9997			
	60	65.36	85.47	0.0200	0.9999			
Men-CP	40	46.08	10.27	0.0048	0.9915	33.17	7.3994	0.9957
	50	49.75	17.01	0.0069	0.9932			
	60	65.36	44.44	0.0104	0.9977			

a *k*, extraction rate constant in g mg^−1^ min^−1^; *C*_s_, equilibrium extraction yield of T-OU in mg g^−1^; *h*, initial extraction rate in mg g^−1^ min^−1^; *E*_a_, activation energy in kJ mol^−1^; *A*_e_, Arrhenius constant in mg g^−1^ min^−1^; *T*, extraction temperature in K.

For the Men–LA system, at 40 °C, the values of *C*_s_, *k*, and *h* were 60.24 mg g^−1^, 0.0049 g mg^−1^ min^−1^, and 17.79 mg g^−1^ min^−1^, respectively, with *R*^2^ = 0.9851. As the temperature increased to 60 °C, both *k* and *h* rose markedly (*k* = 0.0200 g mg^−1^ min^−1^ and *h* = 85.47 mg g^−1^ min^−1^, *R*^2^ = 0.9999). Elevated temperature likely lowered solvent viscosity and promoted the release of compounds from the plant matrix.

In the case of the Men–CP system, at 40 °C, the parameters *C*_s_, *k*, and *h* were 46.08 mg g^−1^, 0.0048 g mg^−1^.min^−1^, and 10.27 mg g^−1^ min^−1^, respectively, with *R*^2^ = 0.9915. At 60 °C, *k* increased to 0.0104 g mg^−1^ min^−1^ and *h* reached 44.44 mg g^−1^ min^−1^ (*R*^2^ = 0.9977). These values were lower than those observed for Men–LA, suggesting a slower extraction rate due to the higher viscosity and weaker wetting ability of the Men–CP solvent, which may hinder molecular diffusion and solute–solvent interactions.

Arrhenius analysis (ln *k versus* 1/*T*) revealed that the activation energy (*E*_a_) for the Men–LA system was 60.84 kJ mol^−1^ (*R*^2^ = 0.9933), while that for Men–CP was 33.17 kJ mol^−1^ (*R*^2^ = 0.9957). According to conventional interpretation, *E*_a_ values above 40 kJ mol^−1^ indicate solubilization-controlled processes, whereas values between 20 and 40 kJ mol^−1^ represent mixed diffusion–solubilization control. Accordingly, the extraction process in Men–LA was primarily controlled by solubilization, while that in Men–CP involved both diffusion and solubilization mechanisms.

Overall, the second-order kinetic analysis clearly showed that the Men–LA system achieved faster and more efficient triterpenoid extraction than Men–CP. This difference is primarily driven by the lower viscosity and higher polarity of Men–LA, which facilitate stronger molecular interactions and faster mass transfer during ultrasonic extraction. These findings confirm that the composition and molar ratio of HDESs components can serve as an effective tool for tuning extraction kinetics and mechanism, thereby optimizing the recovery of triterpenoids from plant materials.

### 3.5. Recovery of triterpenoids

An essential determinant of the practical viability of any extraction strategy is the efficiency with which bioactive constituents can be recovered from green solvent–based extracts. Efficient recovery not only enables the reuse of green solvents and minimizes waste generation but also enhances the sustainability and economic viability of the approach. Moreover, the ability to selectively recover target compounds from complex HDES matrices is essential to ensure product purity and facilitate downstream processing.

**Fig. 6:**
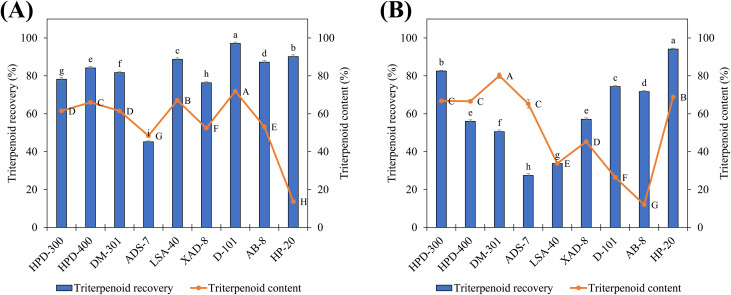
Capability of triterpenoid recovery from Men–LA (A) and Men–CP (B) extracts using SLE based on MRs. Different lettering within the chart signifies statistically meaningful differences among groups at the *p* < 0.05 level.

In this study, triterpenoid recovery from HDESs extracts was conducted using SLE based on MRs. The recovery results of triterpenoids from Men–LA and Men–CP extracts are shown in Fig. 6. Two main parameters, triterpenoid recovery and triterpenoid content, were employed to evaluate the recovery performance of each resin. Here, triterpenoid recovery refers to the percentage of OA and UA recovered after the MRs-based recovery process relative to their initial amounts in the HDESs extract, whereas triterpenoid content refers to the combined percentage of OA and UA in the recovered extract.

For the Men–LA extract, significant variations in triterpenoid recovery were observed among different resins. The recovery yields ranged from 45.19% to 97.11%, while triterpenoid content varied between 48.40% and 71.89%. Among them, D-101 showed the best overall performance, achieving a recovery yield of 97.11% and a triterpenoid content of 71.89%. HP-20 and LSA-40 followed, with recovery yields of 90.18% and 88.72% and triterpenoid contents of 13.68% and 67.08%, respectively. These results indicate that the D-101 resin, composed of a low-polarity styrene divinylbenzene copolymer matrix with a large surface area and small pore diameter, provides favorable conditions for hydrophobic and van der Waals interactions with the pentacyclic backbone of triterpenoids. In contrast, ADS-7 and XAD-8 showed lower recoveries, likely due to their higher polarity, smaller surface area, and larger pore size, which resulted in incomplete adsorption and inefficient desorption within the viscous HDES phase.^49,50^

For the Men–CP extract, the recovery yields ranged from 27.44% to 94.15%, and the triterpenoid content varied from 12.01% to 80.07%. Among them, HP-20 demonstrated the most efficient recovery, achieving the highest yield 94.15% and triterpenoid content of 68.86%. Although DM-301 produced the highest triterpenoid content of 80.07%, its recovery yield was limited to 50.53%. This result reflects the strong compatibility between the hydrophobic surface of HP-20 and the low-polarity Men–CP matrix, which enhances the selective adsorption of triterpenoids. Meanwhile, D-101 achieved moderate recovery 74.36% but low triterpenoid content of 26.43%, indicating nonspecific adsorption caused by competition between triterpenoids and other hydrophobic components, possibly derived from camphor in the HDES phase.

Overall, D-101 and HP-20 were identified as the most effective resins for triterpenoid recovery from Men–LA and Men–CP extracts, respectively. The differences in performance can be attributed to the specific interactions among the HDES matrix, triterpenoids, and the resin surface. Although the underlying processes that drive these interactions require further investigation, the present results emphasize the importance of selecting MRs with physicochemical properties compatible with the polarity of the HDES phase to maximize recovery efficiency and selectivity toward triterpenoids.

The integration of green solvents with MRs offers a sustainable approach to address the non-volatility of these media and to enable the efficient isolation of valuable bioactive constituents from natural materials. Several studies have successfully applied this combined strategy to triterpenoid extraction and purification. For instance, a betaine–lactic acid DESs demonstrated improved effectiveness in recovering triterpenoids from *Prunella vulgaris*, after which the target compounds were separated from the DES phase using D-101 and AB-8 MRs.^51^ Similarly, the combination of choline chloride–lactic acid with AB-8 MRs has proven effective for isolating triterpenoids present in Persimmon leaves.^52^ In addition, bio-based aqueous solvents such as pentane-1,2-diol and hexane-1,2-diol, when coupled with D-941 and LSA-40 resins, have achieved remarkable extraction efficiency for betulinic acid from *Tetracera scandens*.^48^

**Fig. 7:**
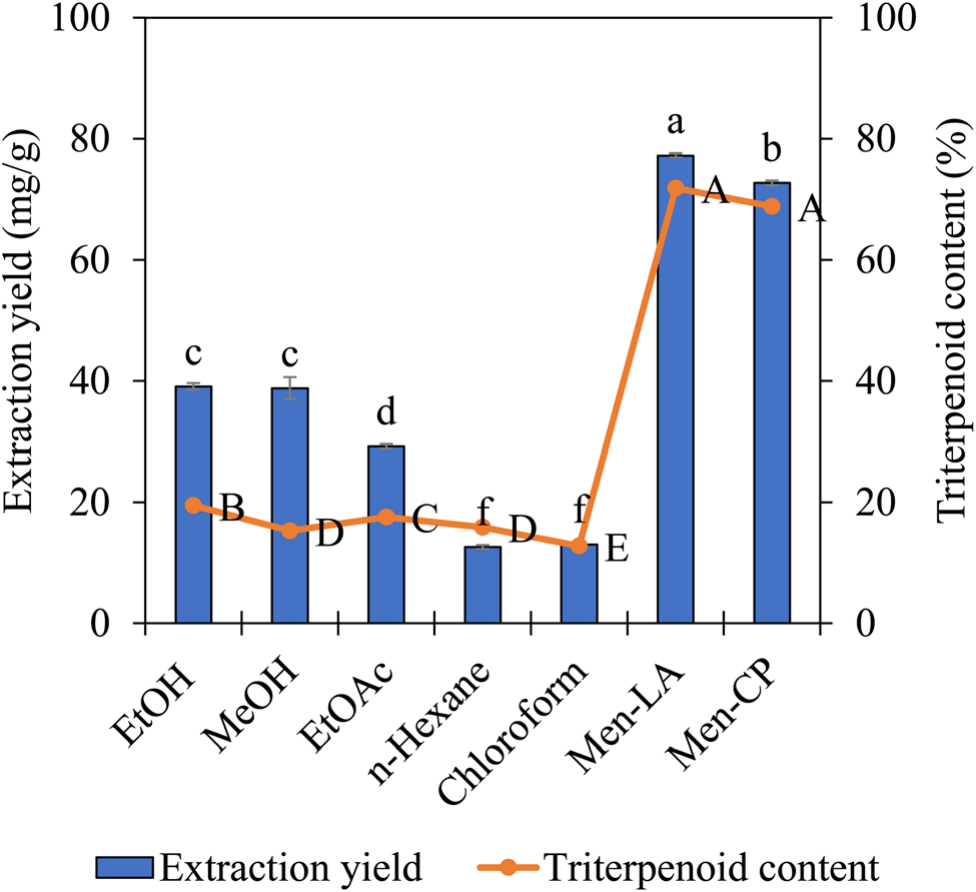
Comparison of extraction yield and triterpenoid content obtained using different extraction solvents, including traditional organic solvents and HDESs. Different lettering within the chart signifies statistically meaningful differences among groups at the *p* < 0.05 level.

In the present study, Men-LA and Men-CP HDESs demonstrated excellent extraction capability toward triterpenoids from Sage. Their combination with MRs not only enabled efficient recovery but also produced extracts with high triterpenoid content. This improvement is clearly illustrated in Fig. 7, which compares the extraction yield and triterpenoid content obtained from different systems. The combined use of HDESs and MRs provided outstanding performance, yielding triterpenoid concentrations ranging from 72.76 to 77.27 mg g^−1^ and triterpenoid contents from 68.86% to 71.89%. In contrast, traditional organic solvents achieved only 12.60–39.10 mg g^−1^ for extraction yield and 12.75–19.44% for triterpenoid content. Consequently, the HDES systems exhibited 1.98–6.13 times higher extraction yields and 3.70–5.64 times higher triterpenoid contents compared with organic solvents. These comparative results highlight the remarkable potential of HDESs for the sustainable extraction of triterpenoids from plant biomass.

### 3.6. Reusability of HDESs and MRs

**Fig. 8:**
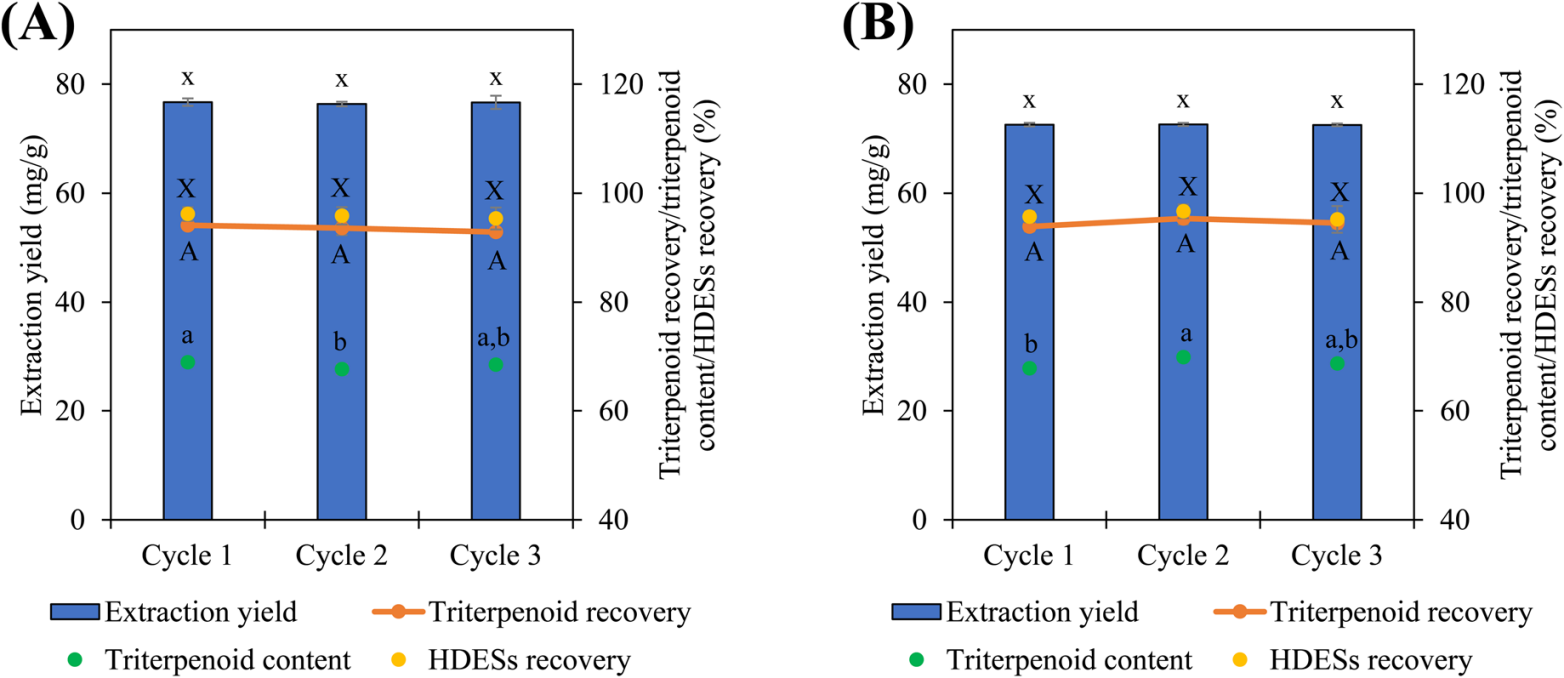
Reusability of HDESs and MRs over three consecutive extraction–recovery cycles for (A) Men–LA and (B) Men–CP systems. Different lettering within the chart signifies statistically meaningful differences among groups at the *p* < 0.05 level.

The reusability of HDESs and MRs was evaluated over three consecutive extraction–recovery cycles to assess the process sustainability and stability. As shown in Fig. 8A and B, both Men–LA and Men–CP systems maintained stable extraction performance throughout three cycles, indicating minimal deterioration in extraction and recovery efficiency.

For the Men–LA system, the extraction yield remained nearly constant (76.76–76.72 mg g^−1^), while triterpenoid recovery showed a slight decrease from 94.08% to 92.84%. The triterpenoid content was maintained within a narrow range (68.95–68.48%), and HDESs recovery remained above 95% in all cycles. For the Men–CP system, a similar stability was observed: the extraction yield fluctuated slightly between 72.62 and 72.65 mg g^−1^, while triterpenoid recovery and content varied within 93.84–95.34% and 67.85–69.89%, respectively. The HDESs recovery also exceeded 95% across all cycles.

The ^1^H NMR spectra of the Men–LA and Men–CP systems, recorded before and after regeneration, demonstrated that the chemical structures of both components remained intact, confirming the excellent structural stability of the HDESs after multiple extraction cycles.

For the Men–LA system, characteristic signals of menthol were observed at *δ* 0.8–0.81, 0.91, 0.93 (three CH_3_ groups), *δ* 3.43–3.47 (CH–OH proton). Lactic acid exhibited diagnostic resonances at *δ* 1.45–1.46 (CH_3_ groups) and *δ* 4.31–4.34 (CH–OH proton). Comparison between the fresh and regenerated solvents revealed negligible changes in chemical shifts and no new peaks in the olefinic (*δ* 4.8–5.2) or aldehydic (*δ* 9–10) regions (Fig. S8 and S9). These results confirm that no dehydration, oxidation, or esterification occurred during use and recovery, and that the hydrogen-bonding network between the hydroxyl group of menthol and the carbonyl group of lactic acid was preserved.

Similarly, the Men–CP spectra exhibited the characteristic signals of menthol at *δ* 0.81–0.82, 0.91–0.91, 0.92–0.93 (CH_3_), and *δ* 3.38–3.42 (CH–OH), along with those of camphor at *δ* 0.84, 0.91, 0.96 (three CH_3_ groups, 9H). After regeneration, all these resonances were retained without the appearance of any new peaks, indicating the absence of decomposition or chemical transformation (Fig. S10 and S11). The minor shift variations compared with Men–LA suggest weaker interactions between menthol and camphor, primarily governed by van der Waals forces, which is consistent with the sterically hindered and rigid bicyclic structure of camphor.

Overall, the excellent overlap between the spectra of fresh and regenerated solvents in both systems indicates that the molar ratios of the components remained unchanged. These findings demonstrate that both Men–LA and Men–CP maintain high structural integrity and strong recyclability, confirming their potential as stable and sustainable green solvents for multicycle triterpenoid extraction.

### 3.7. Comparison of the bioactivities of triterpenoids extracted using green and conventional solvents

Employing green solvents in extraction not only promotes environmental sustainability but also enhances process safety and economic viability. Nevertheless, it is essential to evaluate whether these solvents can maintain or even improve the biological potency of the extracted constituents compared with conventional solvents. In the present work, the bioactivities of triterpenoids obtained using green solvents were systematically compared with those extracted with traditional solvents. Ethanol was selected as the benchmark solvent because it is widely used in natural product extraction and provided the highest extraction yield and triterpenoid content among the tested organic solvents. The comparative analysis focused on three representative biological properties: cytotoxic activity against cancer cells, antioxidant potential, and α-glucosidase inhibitory activity. These evaluations aimed to determine whether green solvents could simultaneously ensure high extraction yields and enhance the pharmacological performance of triterpenoids.

#### 3.7.1. Cytotoxic activity

The cytotoxic effects of triterpenoid extracts from Sage obtained using ethanol (Tri-EtOH) and HDESs (Tri-Men–LA and Tri-Men–CP) were evaluated against a panel of human cancer cell lines, and the results are summarized in Table 3. All extracts exhibited significant cytotoxicity in the low-µg mL^−1^ range, although their potency varied among cell types and solvent systems. Tri-EtOH showed the strongest inhibitory effects on MCF-7 (7.20 ± 0.37 µg mL^−1^), HepG2 (6.07 ± 0.37 µg mL^−1^), and A549 (7.30 ± 0.46 µg mL^−1^) cells, indicating that ethanol efficiently extracted triterpenoids with potent activity against breast, liver, and lung cancer cells. Conversely, Tri-Men–LA displayed greater cytotoxicity toward AGS (7.42 ± 0.53 µg mL^−1^), HT-29 (8.87 ± 0.72 µg mL^−1^), and SK-LU-1 (10.59 ± 0.51 µg mL^−1^) cells, suggesting that the Men–LA solvent system favors triterpenoid constituents effective against gastric, colorectal, and lung cancer cells. In addition, Tri-Men–CP exhibited the most pronounced effects on SW480 (6.31 ± 0.81 µg mL^−1^) and HeLa (6.41 ± 0.48 µg mL^−1^) cells, surpassing both Tri-EtOH and Tri-Men–LA. Regarding the normal HEK-293A cell line, all extracts showed cytotoxic effects in a similar low-µg mL^−1^ range (8.70–9.57 µg mL^−1^), indicating that further purification or fractionation may be necessary to enhance cancer cell selectivity.

**Table 3:** Cytotoxic properties of Sage triterpenoids extracted with organic solvents and HDESs

Cell lines	IC_50_ values (µg mL^−1^)			
	Tri-EtOH	Tri-Men-LA	Tri-Men-CP	Ellipticine
MCF-7	7.20 ± 0.37	11.60 ± 0.67	10.33 ± 0.78	0.37 ± 0.03
HepG2	6.07 ± 0.37	8.63 ± 0.56	6.88 ± 0.41	0.33 ± 0.02
A549	7.30 ± 0.46	9.75 ± 0.66	12.18 ± 0.91	0.39 ± 0.03
AGS	8.34 ± 0.48	7.42 ± 0.53	9.18 ± 0.45	0.34 ± 0.03
HT-29	9.49 ± 0.65	8.87 ± 0.72	10.22 ± 0.52	0.35 ± 0.02
SK-LU-1	10.79 ± 0.23	10.59 ± 0.51	11.56 ± 0.81	0.40 ± 0.03
SW480	7.58 ± 0.45	7.55 ± 0.87	6.31 ± 0.81	0.36 ± 0.03
Hela	7.25 ± 0.26	6.68 ± 0.28	6.41 ± 0.48	0.39 ± 0.02
HEK-293A	9.57 ± 0.46	8.70 ± 0.57	8.43 ± 0.38	0.30 ± 0.02

#### 3.7.2. Antioxidant activity

The antioxidant activity of triterpenoid extracts from Sage obtained using ethanol (Tri-EtOH) and HDESs (Tri-Men–LA and Tri-Men–CP) was assessed, and the corresponding results are summarized in Table 4. All extracts exhibited noticeable antioxidant capacity, with IC_50_ values ranging from 6.60 to 11.81 µg mL^−1^. Among the tested systems, Tri-Men–LA showed the highest antioxidant potential (IC_50_ = 6.60 ± 0.03 µg mL^−1^), followed by Tri-Men–CP (IC_50_ = 7.16 ± 0.26 µg mL^−1^) and Tri-EtOH (IC_50_ = 11.81 ± 0.33 µg mL^−1^). Notably, the antioxidant effect of Tri-Men–LA was even stronger than that of l-ascorbic acid (IC_50_ = 7.79 ± 0.12 µg mL^−1^), highlighting the exceptional efficiency of this HDES system in preserving or enhancing triterpenoid functionality.

**Table 4:** Antioxidant properties of Sage triterpenoids extracted with organic solvents and HDESs

Extract	IC_50_ values (µg mL^−1^)
Tri-EtOH	11.81 ± 0.33
Tri-Men-LA	6.60 ± 0.03
Tri-Men-CP	7.16 ± 0.26
l-Ascorbic acid	7.79 ± 0.12

#### 3.7.3. α-Glucosidase inhibitory activity

The α-glucosidase inhibitory potential of triterpenoids extracted with ethanol (Tri-EtOH), Men–LA (Tri-Men–LA), and Men–CP (Tri-Men–CP) was evaluated, and the results are summarized in Table 5. As shown, the IC_50_ values of Tri-EtOH, Tri-Men–LA, and Tri-Men–CP were 36.71 ± 1.06, 6.73 ± 0.12, and 7.76 ± 0.13 µg mL^−1^, respectively. Both HDES-based extracts exhibited significantly stronger α-glucosidase inhibition than the ethanol extract. In particular, Tri-Men–LA demonstrated the most potent inhibitory effect, with an IC_50_ approximately 5.5-fold lower than that of Tri-EtOH. Moreover, the inhibitory capacities of both HDES extracts were markedly superior to that of the positive control acarbose (IC_50_ = 116.98 ± 7.63 µg mL^−1^), confirming their excellent potential as natural α-glucosidase inhibitors.

**Table 5:** α-Glucosidase inhibitory properties of Sage triterpenoids extracted with organic solvents and HDESs

Extract	IC_50_ values (µg mL^−1^)
Tri-EtOH	36.71 ± 1.06
Tri-Men-LA	6.73 ± 0.12
Tri-Men-CP	7.76 ± 0.13
Acarbose	116.98 ± 7.63

Overall, the bioactivity results demonstrate that the choice of extraction solvent not only determines triterpenoid yield and purity but also significantly shapes the pharmacological profiles of the resulting extracts. For cytotoxic activity, the HDES-derived extracts did not consistently outperform ethanol across all cell lines; instead, they exhibited a distinct pattern of cell-line-specific responses. This variation likely reflects differences in the triterpenoid composition and co-extracted minor constituents enriched by each solvent system, suggesting that HDESs modulate cytotoxicity through shifts in chemical profiles rather than uniform potency enhancement.

In contrast, the antioxidant and α-glucosidase inhibitory activities showed a clear improvement for the HDES extracts compared with ethanol. This enhancement can be associated with the substantially higher triterpenoid content (≈70%) obtained from HDESs relative to Tri-EtOH (19.44%), as well as the possibility that HDESs co-extract auxiliary lipophilic compounds that act synergistically with OA and UA. The superior α-glucosidase inhibition of Tri-Men-LA and Tri-Men-CP further supports this interpretation, indicating that solvent-induced enrichment plays a decisive role in strengthening overall bioactivity.

Although the purification process substantially reduced solvent residues, trace amounts of HDESs may still remain and could partly contribute to the measured antioxidant and α-glucosidase inhibitory activities. Therefore, the enhanced activities of Tri-Men-LA and Tri-Men-CP are interpreted primarily in relation to triterpenoid enrichment and solvent-mediated compositional changes, while a minor influence from residual HDESs cannot be completely excluded. Further studies are warranted to quantify residual HDESs and clarify their specific contribution to the observed activities.

Collectively, these findings highlight that HDESs do not simply increase extraction efficiency but also alter the chemical complexity of the extract in ways that differentially influence specific biological endpoints. Such solvent-dependent variations underscore the importance of tailoring green solvent systems to the intended pharmacological application and illustrate the broader potential of HDES-mediated extraction for generating bioactive fractions with enhanced or specialized biological functions.

### 3.8. Molecular docking study

Among the biological activities exhibited by the triterpenoid-enriched extracts obtained using green solvents, including α-glucosidase inhibition, cytotoxicity, and antioxidant responses, inhibition of α-glucosidase emerged as the dominant effect. The remarkably low IC_50_ values observed for the HDES-based extracts, particularly Tri-Men-LA (6.73 ± 0.12 µg mL^−1^) and Tri-Men-CP (7.76 ± 0.13 µg mL^−1^), highlighted α-glucosidase as a key pharmacological target of these triterpenoids. Therefore, molecular docking simulations were performed to elucidate the molecular basis underlying their inhibitory potential toward α-glucosidase.

As summarized in Table S11, both OA and UA exhibited strong binding affinities of −8.6 and −8.2 kcal mol^−1^, respectively, surpassing that of the reference inhibitor acarbose (−7.7 kcal mol^−1^). These negative binding energies suggest the formation of energetically favorable and stable complexes within the enzyme's active site, supporting their experimentally observed inhibitory potency.

**Fig. 9:**
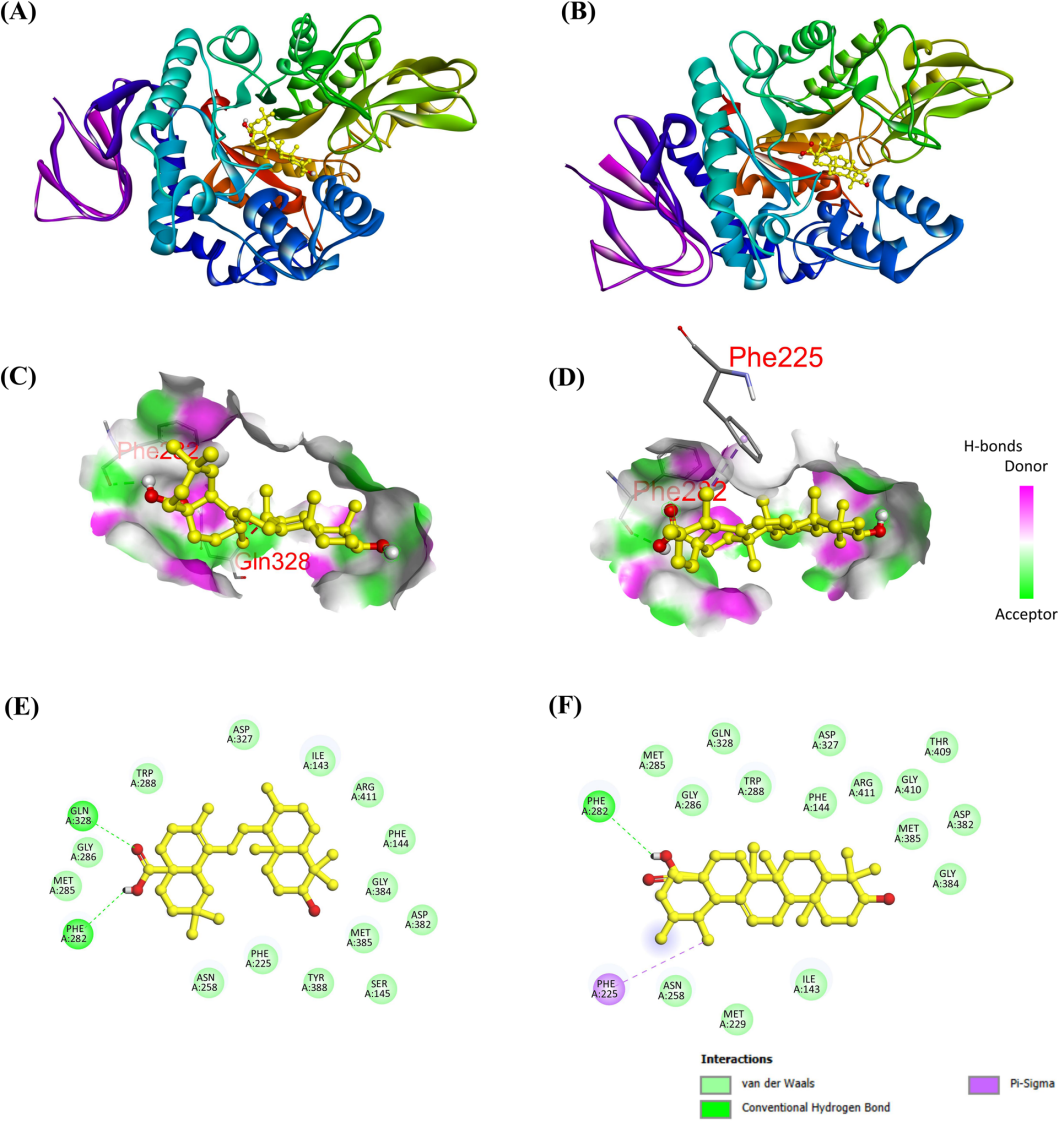
Visualization of the binding interactions between α-glucosidase (PDB: 5ZCC) and OA (left) and UA (right). (A and B) Overall 3D conformations of the enzyme–ligand complexes highlighting the active-site regions. (C and D) Enlarged 3D views showing key residues involved in ligand binding. (E and F) Two-dimensional interaction maps illustrating hydrogen bonding and hydrophobic contacts within the catalytic pocket.

As illustrated in Fig. 9, both triterpenoids were stably accommodated within the hydrophobic catalytic pocket of α-glucosidase through a combination of hydrogen bonding, hydrophobic and van der Waals interactions. For OA, two conventional hydrogen bonds were identified with Gln328 and Phe282, accompanied by a continuous field of van der Waals contacts involving Met285, Gly286, Trp288, Asn258, Arg411, and neighboring residues. These multiple nonpolar interactions formed an extended hydrophobic interface that tightly enclosed the planar pentacyclic skeleton of OA, thereby stabilizing the OA–enzyme complex within the catalytic pocket. In the case of UA, one hydrogen bond with Phe282 and an additional π–*σ* interaction with Phe225 served as anchoring points, while a wide van der Waals contact region extended over Met229, Ile143, Asp327, Gln328, Gly384, Met385, Thr409, and adjacent residues. The coexistence of polar anchoring and broad hydrophobic contacts effectively immobilized the lipophilic backbone of UA within the enzyme's catalytic cavity, reflecting its amphiphilic character and strong compatibility with the nonpolar environment of the pocket.

The consistency between computational binding affinities and experimental IC_50_ values confirms that OA and UA are the principal contributors to the α-glucosidase inhibitory activity of the HDES extracts. In particular, the high triterpenoid content of Tri-Men-LA and Tri-Men-CP is likely responsible for their potent inhibition, possibly through synergistic interactions between OA, UA, and other co-extracted constituents. Further investigations are needed to explore these synergistic mechanisms and to fully clarify the structure–activity relationships governing α-glucosidase inhibition by triterpenoid-rich extracts.

## 4. Conclusion

Hydrophobic deep eutectic solvents (HDESs) demonstrated strong potential as sustainable media for ultrasound-assisted extraction and enrichment of triterpenoids from *Salvia officinalis*. Among the investigated systems, menthol–lactic acid (Men–LA) and menthol–camphor (Men–CP) exhibited superior extraction performance. Response surface optimization further improved triterpenoid yields to 77.27 and 72.76 mg g^−1^ for Men–LA and Men–CP, respectively, corresponding to approximately two-to six-fold higher recoveries than those obtained using conventional organic solvents. Kinetic investigations revealed that triterpenoid extraction followed a second-order model, indicating the combined influence of diffusion and solubilization processes. Complementary computational analyses further demonstrated that favorable hydrogen bonding and van der Waals interactions between HDESs components and triterpenoid molecules promoted solute–solvent stabilization and facilitated mass transfer, thereby providing mechanistic insight into the superior extraction efficiency of the selected systems. The practical feasibility of the proposed approach was demonstrated through integration with macroporous resins, enabling triterpenoid recoveries of 94–97%, product purities approaching 70%, and HDESs recyclability exceeding 95%. Moreover, regenerated HDESs retained their structural integrity according to ^1^H NMR analyses, confirming solvent stability during repeated reuse cycles. Beyond extraction efficiency, HDESs-derived triterpenoid fractions exhibited enhanced antioxidant and α-glucosidase inhibitory activities relative to ethanol extracts and displayed distinct cytotoxic profiles across different cancer cell lines. Molecular docking further supported the stable interaction of oleanolic acid and ursolic acid with the α-glucosidase active site, providing molecular-level evidence for their inhibitory activity. Taken together, these findings establish HDESs as recyclable, mechanistically understood, and high-performance green solvents for ultrasound-assisted triterpenoid extraction and enrichment, while offering a rational framework for the future development of sustainable extraction systems targeting lipophilic natural bioactives.

## Author contributions

Nhan Trong Le: methodology, investigation, writing – original draft preparation; The-Huan Tran: investigation, software, writing – review and editing; Thi Thi Thi Nguyen: investigation, formal analysis; Thao Thi Do: investigation, formal analysis; Hoai Thi Nguyen: conceptualization, methodology, writing - review and editing, supervision.

## Conflicts of interest

There are no conflicts to declare.

## Supplementary Material

RA-016-D6RA03456J-s001

## Data Availability

Data will be made available upon request. Supplementary information (SI) is available. See DOI: https://doi.org/10.1039/d6ra03456j.
