# Supplementary material for: Hydrophobic deep eutectic solvents as sustainable media for ultrasound-assisted extraction of triterpenoids: a mechanistic and experimental study on Salvia officinalis L
Source: RSC Adv. 2026 Jul 3;16(35):35862–79. doi: 10.1039/d6ra03456j (PMC13330174; doi:10.1039/d6ra03456j)
Supplement: RA-016-D6RA03456J-s001 [file RA-016-D6RA03456J-s001.pdf]

Supplementary Information (SI) for RSC Advances.
This journal is © The Royal Society of Chemistry 2026

Supplementary material

Hydrophobic Deep Eutectic Solvents as Sustainable Media for Ultrasound-Assisted Extraction of Triterpenoids: A Mechanistic and Experimental Study on *Salvia officinalis* L.

Nhan Trong Le[a], The-Huan Tran[a], Thi Thi Thi Nguyen[a], Thao Thi Do[b], and Hoai Thi Nguyen[*a]

[a] Faculty of Pharmacy, Hue University of Medicine and Pharmacy, Hue University, Hue City, Vietnam.
[b] Institute of Biology, Vietnam Academy of Science and Technology, Hanoi, Vietnam.
* Correspondence: Hoai Thi Nguyen; e-mail: nthoai@hueuni.edu.vn; Faculty of Pharmacy, Hue University of Medicine and Pharmacy, Hue University, Hue City, Vietnam.

Table of contents	Page
**Table S1.** Chemicals and reagents used in this study, including CAS numbers, purities, and commercial suppliers.	**2**
**Table S2.** Independent variables and corresponding coded levels employed for the optimization of triterpenoid extraction using the Men–LA HDESs.	**3**
**Table S3.** Independent variables and corresponding coded levels employed for the optimization of triterpenoid extraction using the Men–CP HDESs.	**3**
**Table S4.** Extraction yields of OA, UA, and T-OU obtained using different HDESs and relative differences compared with the selected HDESs (Men–LA and Men–CP).	**3**
**Table S5.** Experimental design matrix based on the Box–Behnken design and corresponding extraction yields of triterpenoid obtained using the Men–LA HDESs.	**4**
**Table S6.** Experimental design matrix based on the Box–Behnken design and corresponding extraction yields of triterpenoid obtained using the Men–CP HDESs.	**5**
**Table S7.** ANOVA results for the triterpenoid extraction process using Men–LA HDESs.	**6**
**Table S8.** ANOVA results for the triterpenoid extraction process using Men–CP HDESs.	**7**
**Table S9.** Relative deviations between experimental and predicted values for triterpenoid extraction in 29 Box–Behnken design runs using the Men–LA HDESs.	**8**
**Table S10.** Relative deviations between experimental and predicted values for triterpenoid extraction in 29 Box–Behnken design runs using the Men–CP HDESs.	**9**
**Table S11.** Binding affinities of oleanolic acid, ursolic acid, and acarbose toward α-glucosidase (PDB: 5ZCC).	**9**
**Figure S1.** HPLC chromatograms of oleanolic acid (OA) and ursolic acid (UA) detected at 205 nm: (A) reference standards and (B) sample extract.	**10**
**Figure S2.** Correlation between predicted and experimental values for triterpenoid extraction using the Men–LA HDESs.	**11**
**Figure S3.** Correlation between predicted and experimental values for triterpenoid extraction using the Men–CP HDESs.	**11**
**Figure S4.** Second-order kinetic models (t/C_t vs t) for triterpenoid extraction from Sage using Men–LA HDES at 40°C (A), 50°C (B), and 60°C (C).	**12**
**Figure S5.** Second-order kinetic models (t/C_t vs t) for triterpenoid extraction from Sage using Men–CP HDES at 40°C (A), 50°C (B), and 60°C (C).	**12**
**Figure S6.** Arrhenius plot derived from the second-order kinetic model for triterpenoid extraction from Sage using Men–LA HDES	**12**
**Figure S7.** Arrhenius plot derived from the second-order kinetic model for triterpenoid extraction from Sage using Men–CP HDES.	**12**
**Figure S8.** ^{1}H NMR spectra of fresh Men-LA.	**13**
**Figure S9.** ^{1}H NMR spectra of recycled Men-LA.	**14**
**Figure S10.** ^{1}H NMR spectra of fresh Men-CP.	**15**
**Figure S11.** ^{1}H NMR spectra of recycled Men-CP.	**16**
**Figure S12.** Stacked ^{1}H NMR spectra of fresh (red) and recycled (green) Men–LA HDESs, including full-spectrum and expanded views for direct comparison of spectral profiles.	**17**
**Figure S13.** Stacked ^{1}H NMR spectra of fresh (red) and recycled (green) Men–CP HDESs, including full-spectrum and expanded views for direct comparison of spectral profiles.	**18**

Table S1. Chemicals and reagents used in this study, including CAS numbers, purities, and commercial suppliers.

No.	Compound	CAS number	Purity (wt%)	Supplier
1	Oleanolic acid	508-02-1	97	Macklin
2	Ursolic acid	77-52-1	98	Macklin
3	Propionic acid	79-09-4	≥99.5	Macklin
4	Octanoic acid	124-07-2	99	Macklin
5	Pyruvic acid	127-17-3	98	Macklin
6	Butyric acid	107-92-6	99	Macklin
7	Nonanoic acid	12-05-0	96	Macklin
8	Capric acid	334-48-5	98	Macklin
9	Lauric acid	143-07-7	98	Macklin
10	Menthol	1490-04-6	98	Macklin
11	Thymol	89-83-8	98	Macklin
12	Camphor	76-22-2	99	Macklin
13	Acetic acid	64-19-7	≥99.5	Xilong
14	Lactic acid	79-33-4	85.5-90	Xilong
15	Ethanol	64-17-5	99.7	Xilong
16	Methanol	67-56-1	99.5	Xilong
17	*n*-Hexane	110-54-3	>97	Xilong
18	Ethyl acetate	141-78-6	99.5	Xilong
19	Chloroform	67-66-3	≥99	Xilong
20	Sodium hydroxide	1310-73-2	≥96	Xilong
21	Hydrochloric acid	7647-01-0	36-38	Xilong
22	Tris base	77-86-1	99.8	Sigma-Aldrich
23	Sulforhodamine B	3520-42-1	75	Sigma-Aldrich
24	Dimethyl sulphoxide	67-68-5	≥99	Sigma-Aldrich
25	Enzyme α-glucosidase	9001-42-7	-	Sigma-Aldrich
26	p-Nitrophenyl-α-D-glucopyranoside	3767-28-0	≥99	Sigma-Aldrich
27	Acarbose	56180-94-0	≥95	Sigma-Aldrich
28	2,2-Diphenyl-1-picrylhydrazyl	1898-66-4	-	Sigma-Aldrich
29	L-ascorbic acid	50-81-7	99	Sigma-Aldrich
30	Sodium bicarbonate	144-55-8	≥99.5	Sigma-Aldrich
31	Trichloroacetic acid	76-03-9	≥99	Sigma-Aldrich
32	Ellipticine	519-23-3	≥95	Sigma-Aldrich
33	Dulbecco's modified Eagle's medium	12430047	-	Thermo Fisher Scientific
34	L-glutamine	25030081	-	Thermo Fisher Scientific
35	Fetal bovine serum	A5670501	-	Thermo Fisher Scientific

Table S2. Independent variables and corresponding coded levels employed for the optimization of triterpenoid extraction using the Men–LA HDESs.

Independent variables	Coded symbols	Coded levels		
		-1	0	+1
LA-to-Men ratio (mol/mol)	A	0.5	2.5	4.5
Liquid-solid ratio (mL/g)	B	10	25	40
Extraction time (min)	C	10	35	60
Extraction temperature (°C)	D	30	50	70

Table S3. Independent variables and corresponding coded levels employed for the optimization of triterpenoid extraction using the Men–CP HDESs.

Independent variables	Coded symbols	Coded levels		
		-1	0	+1
Men-to-CP ratio (mol/mol)	A	1	2.5	4
Liquid-solid ratio (mL/g)	B	20	35	50
Extraction time (min)	C	10	35	60
Extraction temperature (°C)	D	30	50	70

Table S4. Extraction yields of OA, UA, and T-OU obtained using different HDESs and relative differences compared with the selected HDESs (Men–LA and Men–CP).

Solvents	Extraction yields (mg/g)			Difference vs selected HDESs (%)	
	OA	UA	T-OU	Men-LA	Men-CP
Men-AA	14.37	27.52	41.88	-32.39	-32.05
Men-LA	22.81	39.13	61.94	0	0.49
Men-PrA	15.19	28.46	43.65	-29.53	-29.19
Men-PyA	16.19	29.33	45.52	-26.51	-26.15
Men-BA	15.19	28.20	43.39	-29.95	-29.60
Men-OA	13.37	23.81	37.18	-39.98	-39.68
Men-NA	14.26	25.24	39.51	-36.22	-35.91
Men-CA	13.93	22.58	36.51	-41.06	-40.77
Men-CP	22.35	39.30	61.64	-0.49	0
Men-TM	20.62	32.64	53.27	-14.01	-13.59
Men-LaA	15.44	25.43	40.87	-34.02	-33.70
LaA-OA	19.18	33.10	52.28	-15.60	-15.19
LaA-NA	17.40	29.64	47.04	-24.07	-23.69
LaA-CA	19.84	33.76	53.60	-13.46	-13.04
EtOH	14.24	24.86	39.10	-36.88	-36.58
MeOH	14.05	24.75	38.81	-37.35	-37.04
EtOAc	10.89	18.32	29.21	-52.84	-52.61
n-Hexane	4.81	7.80	12.60	-79.66	-79.56
Chloroform	4.68	8.35	13.03	-78.97	-78.87

OA: oleanolic acid, UA: ursolic acid; T-OU: total oleanolic acid and ursolic acid. Relative difference (%) was calculated based on T-OU values using Men–LA or Men–CP as the selected HDESs references according to the equation: Relative difference (%) = $[(T\text{-}OU_i - T\text{-}OU_{selected})/T\text{-}OU_{selected}] \times 100$, where $T\text{-}OU_i$ represents the T-OU yield obtained using a given HDESs, and $T\text{-}OU_{selected}$ represents the T-OU yield obtained using the selected HDESs (Men–LA or Men–CP).

Table S5. Experimental design matrix based on the Box–Behnken design and corresponding extraction yields of triterpenoid obtained using the Men–LA HDESs.

Run	A	B	C	D	Extraction yields (mg/g)		
					OA	UA	T-OU
1	2.5	10	35	70	18.02	30.11	48.13
2	0.5	10	35	50	17.34	29.95	47.28
3	2.5	25	35	50	24.92	41.05	65.97
4	2.5	25	35	50	22.48	40.98	63.45
5	2.5	25	10	30	16.11	26.02	42.13
6	2.5	25	60	30	20.43	28.70	49.13
7	2.5	10	35	30	14.15	21.33	35.48
8	0.5	25	60	50	24.75	40.40	65.15
9	4.5	25	35	30	15.65	26.78	42.42
10	0.5	25	10	50	21.91	36.67	58.59
11	4.5	10	35	50	18.11	30.49	48.60
12	2.5	25	35	50	23.67	41.50	65.16
13	2.5	25	10	70	21.85	38.57	60.42
14	2.5	40	35	30	21.19	34.30	55.49
15	2.5	10	60	50	16.58	28.91	45.49
16	2.5	40	35	70	27.40	47.41	74.82
17	2.5	25	35	50	24.16	43.74	67.90
18	4.5	25	10	50	20.21	34.87	55.08
19	4.5	25	35	70	27.39	46.71	74.10
20	2.5	40	60	50	27.45	46.75	74.20
21	2.5	25	35	50	23.35	41.43	64.79
22	0.5	40	35	50	26.89	45.29	72.18
23	2.5	10	10	50	16.62	30.52	47.13
24	2.5	25	60	70	25.11	41.76	66.87
25	4.5	40	35	50	26.29	44.89	71.19
26	0.5	25	35	30	20.93	35.62	56.55
27	0.5	25	35	70	21.49	37.09	58.59
28	4.5	25	60	50	22.76	39.64	62.40
29	2.5	40	10	50	22.86	37.73	60.59

A: LA-to-Men ratio (mol/mol), B: liquid-to-solid ratio (mL/g), C: extraction time (min), D: extraction temperature (°C), OA: oleanolic acid, UA: ursolic acid; T-OU: total oleanolic acid and ursolic acid.

Table S6. Experimental design matrix based on the Box–Behnken design and corresponding extraction yields of triterpenoid obtained using the Men–CP HDESs.

Run	A	B	C	D	Extraction yields (mg/g)		
					OA	UA	T-OU
1	2.5	20	35	70	23.22	37.38	60.60
2	2.5	50	60	50	28.21	39.53	67.74
3	2.5	50	10	50	20.01	29.62	49.63
4	1	50	35	50	20.45	31.42	51.87
5	2.5	35	60	30	27.53	45.60	73.13
6	4	50	35	50	22.78	35.63	58.42
7	2.5	50	35	70	24.60	40.31	64.91
8	2.5	50	35	30	24.86	38.36	63.22
9	4	20	35	50	18.49	31.08	49.57
10	4	35	10	50	20.33	32.53	52.86
11	2.5	20	35	30	23.50	37.87	61.36
12	2.5	35	35	50	26.56	43.93	70.49
13	1	35	35	70	23.54	40.51	64.06
14	1	35	35	30	22.34	36.56	58.91
15	2.5	20	10	50	22.55	36.86	59.40
16	2.5	20	60	50	19.93	30.04	49.96
17	2.5	35	35	50	26.89	42.99	69.88
18	2.5	35	35	50	27.23	45.05	72.27
19	1	35	60	50	20.62	35.07	55.69
20	2.5	35	10	70	26.95	40.39	67.34
21	1	20	35	50	19.87	29.35	49.23
22	1	35	10	50	22.95	34.65	57.61
23	4	35	60	50	25.08	39.77	64.85
24	2.5	35	10	30	24.65	36.19	60.83
25	2.5	35	35	50	26.63	45.22	71.85
26	4	35	35	70	23.85	40.92	64.77
27	2.5	35	35	50	26.25	42.18	68.43
28	4	35	35	30	24.88	42.60	67.48
29	2.5	35	60	70	27.21	38.71	65.91

A: Men-to-CP ratio (mol/mol), B: liquid-to-solid ratio (mL/g), C: extraction time (min), D: extraction temperature (°C), OA: oleanolic acid, UA: ursolic acid; T-OU: total oleanolic acid and ursolic acid.

Table S7. ANOVA results for the triterpenoid extraction process using Men–LA HDESs.

Source	Sum of Squares	df	Mean Square	F-value	p-value	
Model	3265.28	14	233.23	120.71	< 0.0001	significant
A	1.72	1	1.72	0.8902	0.3614	
B	1549.13	1	1549.13	801.75	< 0.0001	
C	128.63	1	128.63	66.57	< 0.0001	
D	862.23	1	862.23	446.25	< 0.0001	
AB	1.34	1	1.34	0.6941	0.4188	
AC	0.1433	1	0.1433	0.0741	0.7894	
AD	219.57	1	219.57	113.64	< 0.0001	
BC	58.15	1	58.15	30.09	< 0.0001	
BD	11.15	1	11.15	5.77	0.0308	
CD	0.0746	1	0.0746	0.0386	0.8471	
A^2	5.01	1	5.01	2.59	0.1297	
B^2	150.74	1	150.74	78.01	< 0.0001	
C^2	103.62	1	103.62	53.63	< 0.0001	
D^2	306.91	1	306.91	158.84	< 0.0001	
Residual	27.05	14	1.93			
Lack of Fit	16.27	10	1.63	0.6034	0.7646	not significant
Pure Error	10.78	4	2.70			
Cor Total	3292.33	28				

$R^2 = 0.9918$ R^2 adj = 0.9836 R^2 pred = 0.9664

A: LA-to-Men ratio (mol/mol), B: liquid-to-solid ratio (mL/g), C: extraction time (min), D: extraction temperature (°C)

Table S8. ANOVA results for the triterpenoid extraction process using Men–CP HDESs.

Source	Sum of Squares	df	Mean Square	F-value	p-value	
Model	1511.15	14	107.94	73.97	< 0.0001	significant
A	35.33	1	35.33	24.21	0.0002	
B	54.92	1	54.92	37.64	< 0.0001	
C	73.08	1	73.08	50.09	< 0.0001	
D	0.5951	1	0.5951	0.4078	0.5334	
AB	9.62	1	9.62	6.59	0.0224	
AC	48.41	1	48.41	33.18	< 0.0001	
AD	15.43	1	15.43	10.57	0.0058	
BC	189.75	1	189.75	130.04	< 0.0001	
BD	1.51	1	1.51	1.03	0.3270	
CD	47.07	1	47.07	32.26	< 0.0001	
A^2	452.65	1	452.65	310.21	< 0.0001	
B^2	588.67	1	588.67	403.43	< 0.0001	
C^2	140.00	1	140.00	95.95	< 0.0001	
D^2	10.95	1	10.95	7.50	0.0160	
Residual	20.43	14	1.46			
Lack of Fit	10.82	10	1.08	0.4503	0.8604	not significant
Pure Error	9.61	4	2.40			
Cor Total	1531.57	28				
	R^2 = 0.9867		R^2 adj = 0.9733		R^2 pred = 0.9495	

A: Men-to-CP ratio (mol/mol), B: liquid-to-solid ratio (mL/g), C: extraction time (min), D: extraction temperature (°C)

Table S9. Relative deviations between experimental and predicted values for triterpenoid extraction in 29 Box–Behnken design runs using the Men–LA HDESs.

Run	A	B	C	D	T-OU extraction yields (mg/g)

					Experimental	Predicted	Relative deviation
1	2.5	10	35	70	48.13	49.20	-2.17
2	0.5	10	35	50	47.28	48.19	-1.89
3	2.5	25	35	50	65.97	65.45	0.79
4	2.5	25	35	50	63.45	65.45	-3.06
5	2.5	25	10	30	42.13	42.69	-1.31
6	2.5	25	60	30	49.13	49.51	-0.77
7	2.5	10	35	30	35.48	35.59	-0.31
8	0.5	25	60	50	65.15	64.04	1.73
9	4.5	25	35	30	42.42	41.43	2.39
10	0.5	25	10	50	58.59	57.87	1.24
11	4.5	10	35	50	48.60	48.59	0.02
12	2.5	25	35	50	65.16	65.45	-0.44
13	2.5	25	10	70	60.42	59.92	0.83
14	2.5	40	35	30	55.49	54.97	0.95
15	2.5	10	60	50	45.49	44.74	1.68
16	2.5	40	35	70	74.82	75.26	-0.58
17	2.5	25	35	50	67.90	65.45	3.74
18	4.5	25	10	50	55.08	56.74	-2.93
19	4.5	25	35	70	74.10	73.20	1.23
20	2.5	40	60	50	74.20	75.09	-1.19
21	2.5	25	35	50	64.79	65.45	-1.01
22	0.5	40	35	50	72.18	72.07	0.15
23	2.5	10	10	50	47.13	45.81	2.88
24	2.5	25	60	70	66.87	66.19	1.03
25	4.5	40	35	50	71.19	70.16	1.47
26	0.5	25	35	30	56.55	57.01	-0.81
27	0.5	25	35	70	58.59	59.14	-0.93
28	4.5	25	60	50	62.40	63.66	-1.98
29	2.5	40	10	50	60.59	60.91	-0.53

A: LA-to-Men ratio (mol/mol), B: liquid-to-solid ratio (mL/g), C: extraction time (min), D: extraction temperature (°C)

Table S10. Relative deviations between experimental and predicted values for triterpenoid extraction in 29 Box–Behnken design runs using the Men–CP HDESs.

Run	A	B	C	D	T-OU extraction yields (mg/g)		
					Experimental	Predicted	Relative deviation
1	2.5	20	35	70	60.60	59.83	1.29
2	2.5	50	60	50	67.74	67.91	-0.25
3	2.5	50	10	50	49.63	49.20	0.87
4	1	50	35	50	51.87	51.58	0.56
5	2.5	35	60	30	73.13	72.91	0.30
6	4	50	35	50	58.42	58.11	0.53
7	2.5	50	35	70	64.91	65.33	-0.64
8	2.5	50	35	30	63.22	63.66	-0.69
9	4	20	35	50	49.57	50.73	-2.29
10	4	35	10	50	52.86	53.36	-0.94
11	2.5	20	35	30	61.36	60.61	1.24
12	2.5	35	35	50	70.49	70.59	-0.14
13	1	35	35	70	64.06	64.00	0.09
14	1	35	35	30	58.91	59.63	-1.21
15	2.5	20	10	50	59.40	58.69	1.21
16	2.5	20	60	50	49.96	49.85	0.22
17	2.5	35	35	50	69.88	70.59	-1.01
18	2.5	35	35	50	72.27	70.59	2.38
19	1	35	60	50	55.69	54.86	1.51
20	2.5	35	10	70	67.34	68.42	-1.58
21	1	20	35	50	49.23	50.40	-2.32
22	1	35	10	50	57.61	56.88	1.28
23	4	35	60	50	64.85	65.25	-0.61
24	2.5	35	10	30	60.83	61.12	-0.47
25	2.5	35	35	50	71.85	70.59	1.78
26	4	35	35	70	64.77	63.51	1.98
27	2.5	35	35	50	68.43	70.59	-3.06
28	4	35	35	30	67.48	66.99	0.73
29	2.5	35	60	70	65.91	66.50	-0.89

A: Men-to-CP ratio (mol/mol), B: liquid-to-solid ratio (mL/g), C: extraction time (min), D: extraction temperature (°C)

Table S11. Binding affinities of oleanolic acid, ursolic acid, and acarbose toward α-glucosidase (PDB: 5ZCC).

Compounds	Binding affinity (kcal/mol)
Oleanolic acid	-8.6
Ursolic acid	-8.2
Acarbose	-7.7

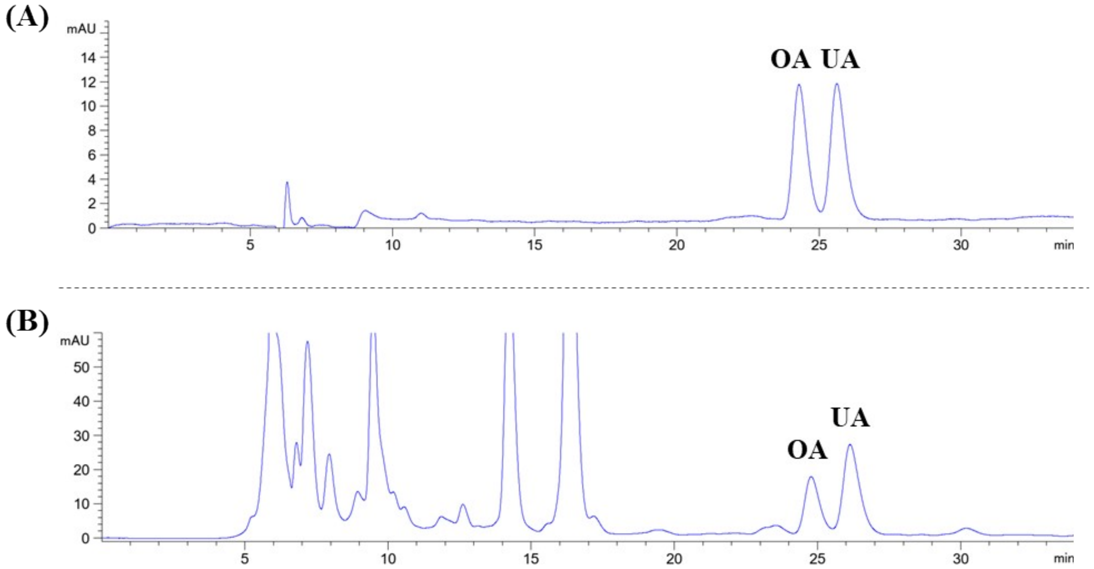

Figure S1. HPLC chromatograms of oleanolic acid (OA) and ursolic acid (UA) detected at 205 nm: (A) reference standards and (B) sample extract.

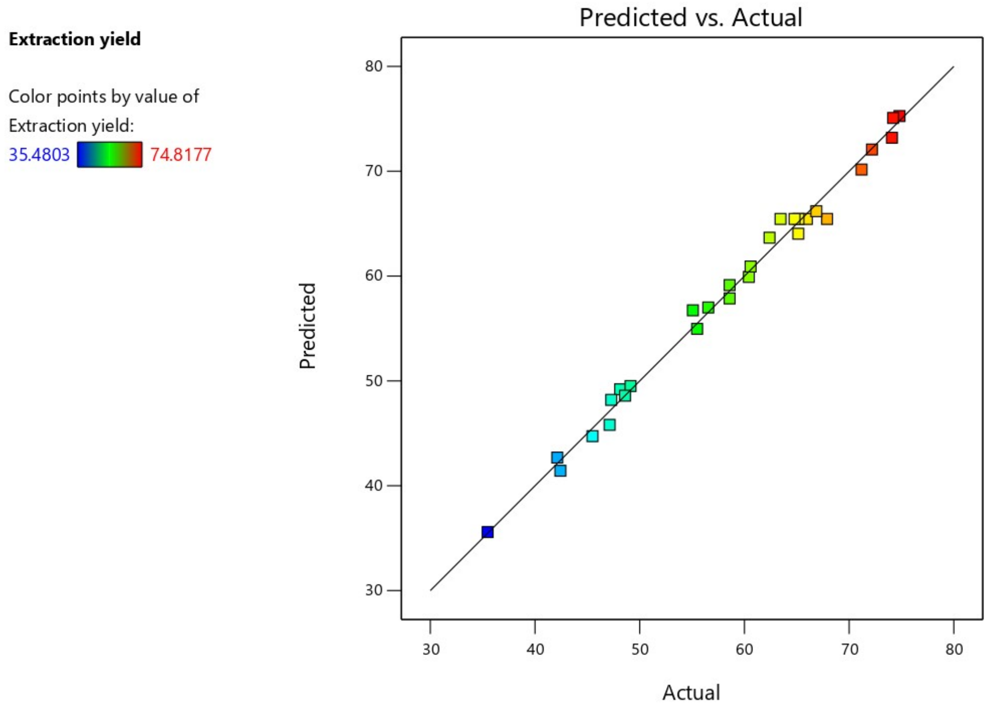

Figure S2. Correlation between predicted and experimental values for triterpenoid extraction using the Men–LA HDESs.

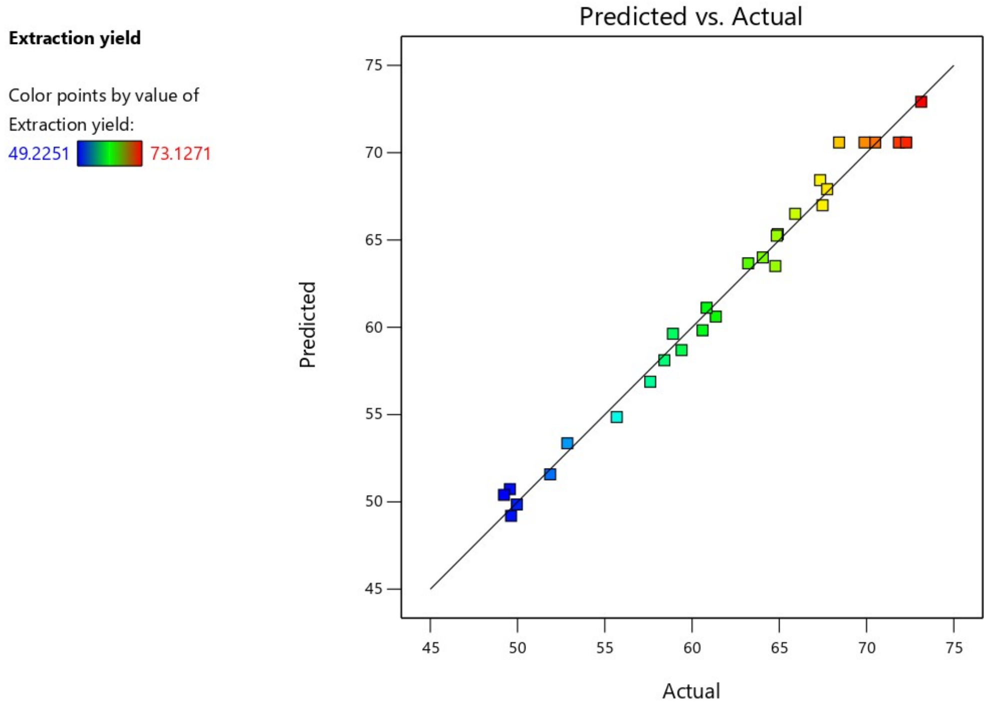

Figure S3. Correlation between predicted and experimental values for triterpenoid extraction using the Men–CP HDESs.

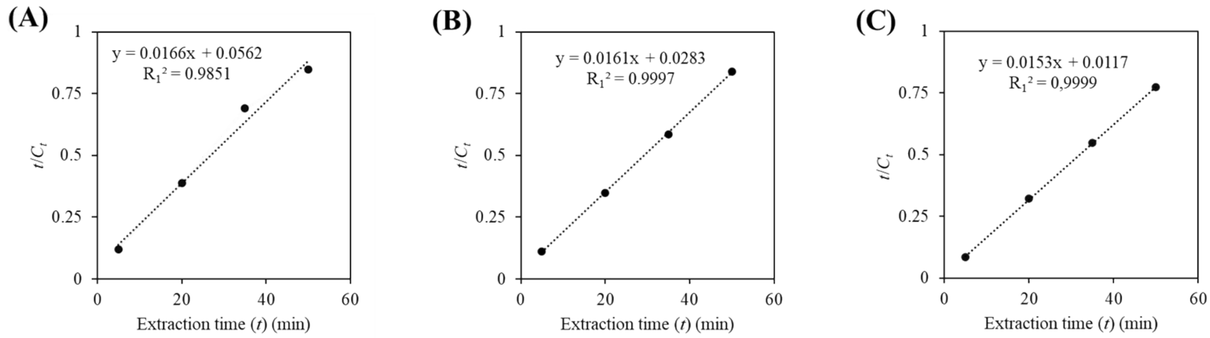

Figure S4. Second-order kinetic models (t/C_t vs t) for triterpenoid extraction from Sage using Men–LA HDES at 40°C (A), 50°C (B), and 60°C (C).

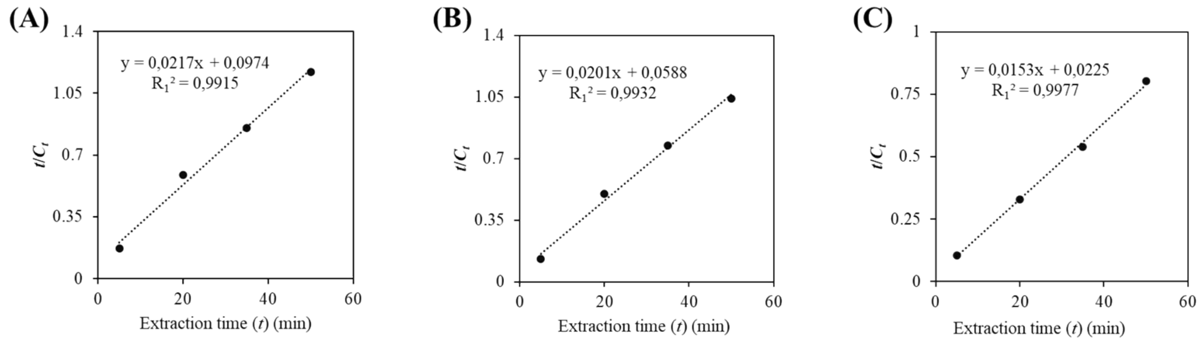

Figure S5. Second-order kinetic models (t/C_t vs t) for triterpenoid extraction from Sage using Men–CP HDES at 40°C (A), 50°C (B), and 60°C (C).

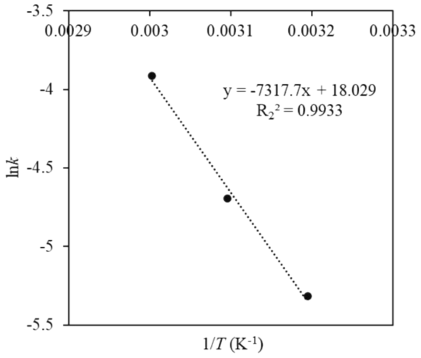

Figure S6. Arrhenius plot derived from the second-order kinetic model for triterpenoid extraction from Sage using Men–LA HDES.

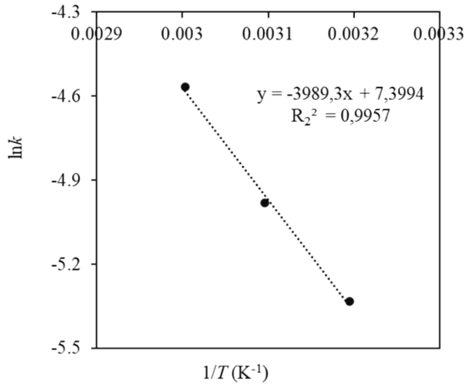

Figure S7. Arrhenius plot derived from the second-order kinetic model for triterpenoid extraction from Sage using Men–CP HDES.

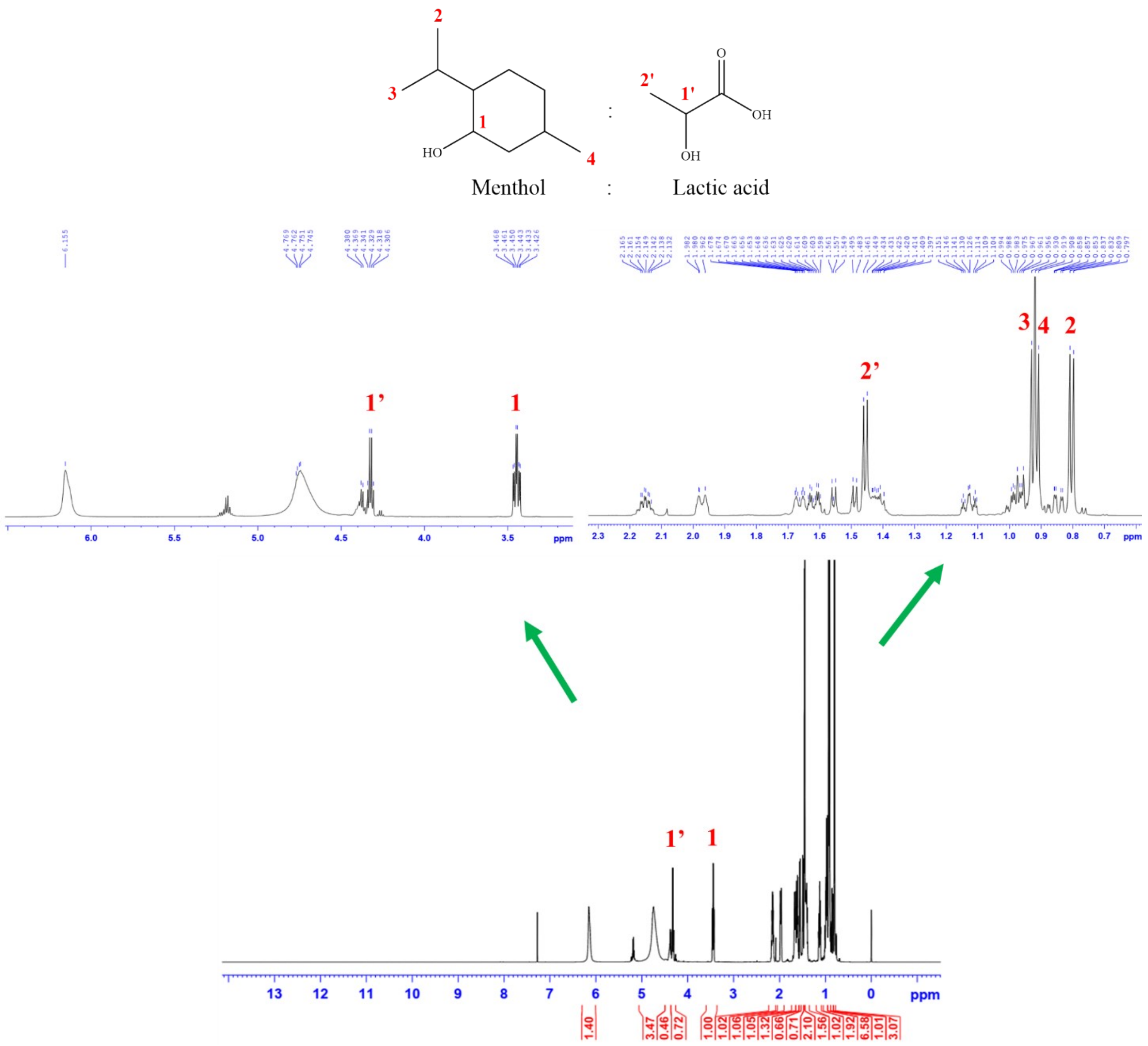

Figure S8. ^{1}H NMR spectra of fresh Men-LA.

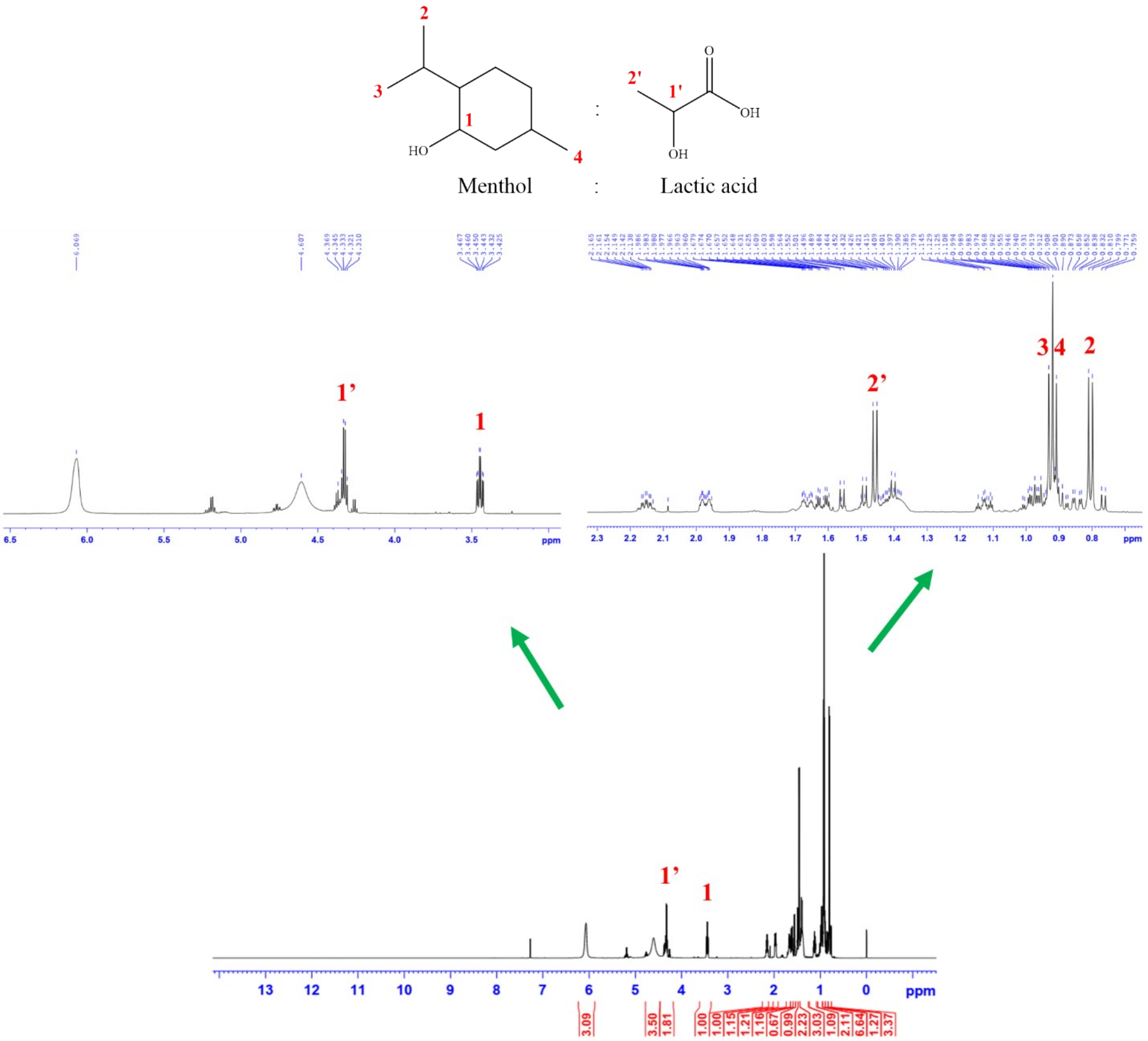

Figure S9. ^{1}H NMR spectra of recycled Men-LA.

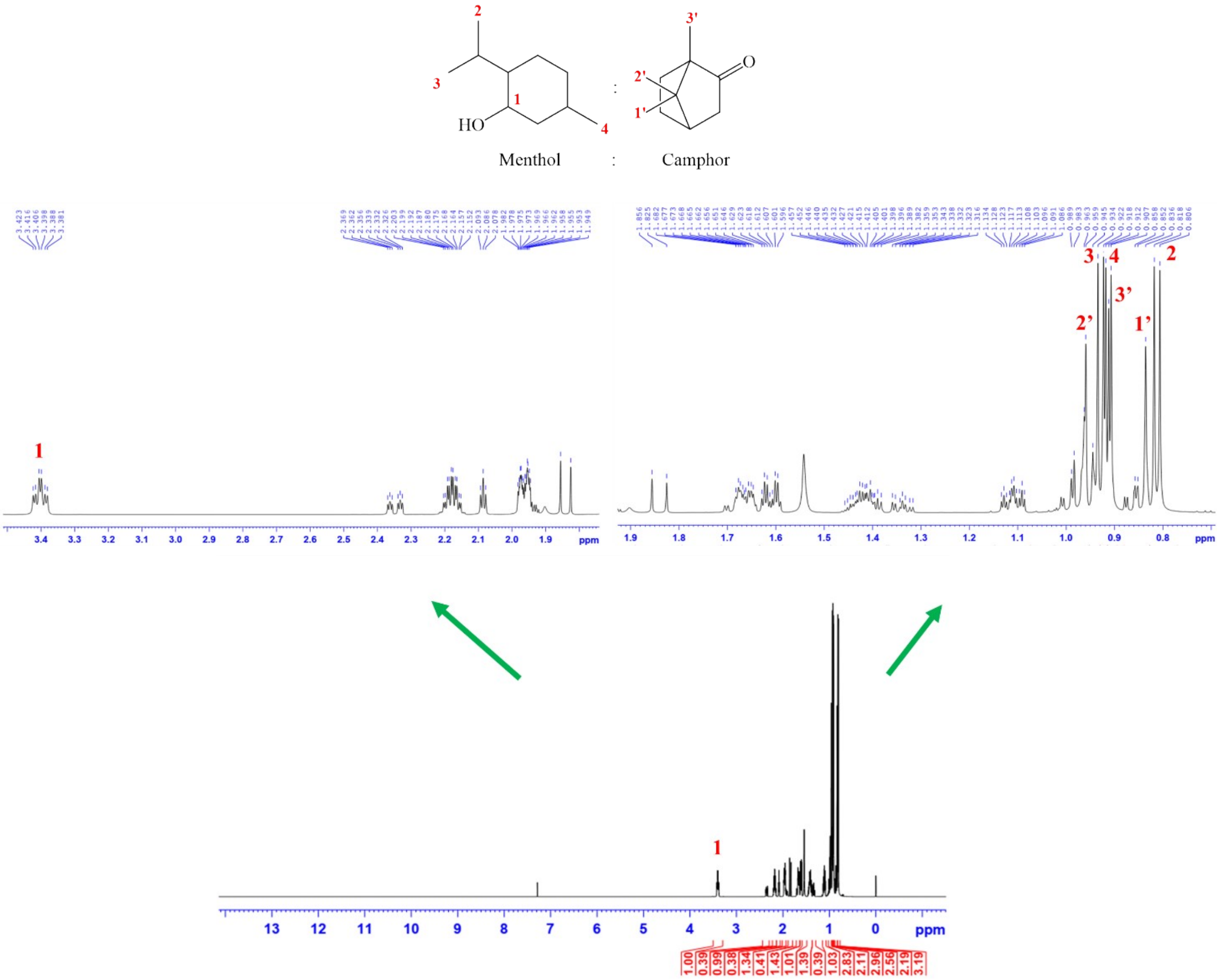

Figure S10. ^{1}H NMR spectra of fresh Men-CP.

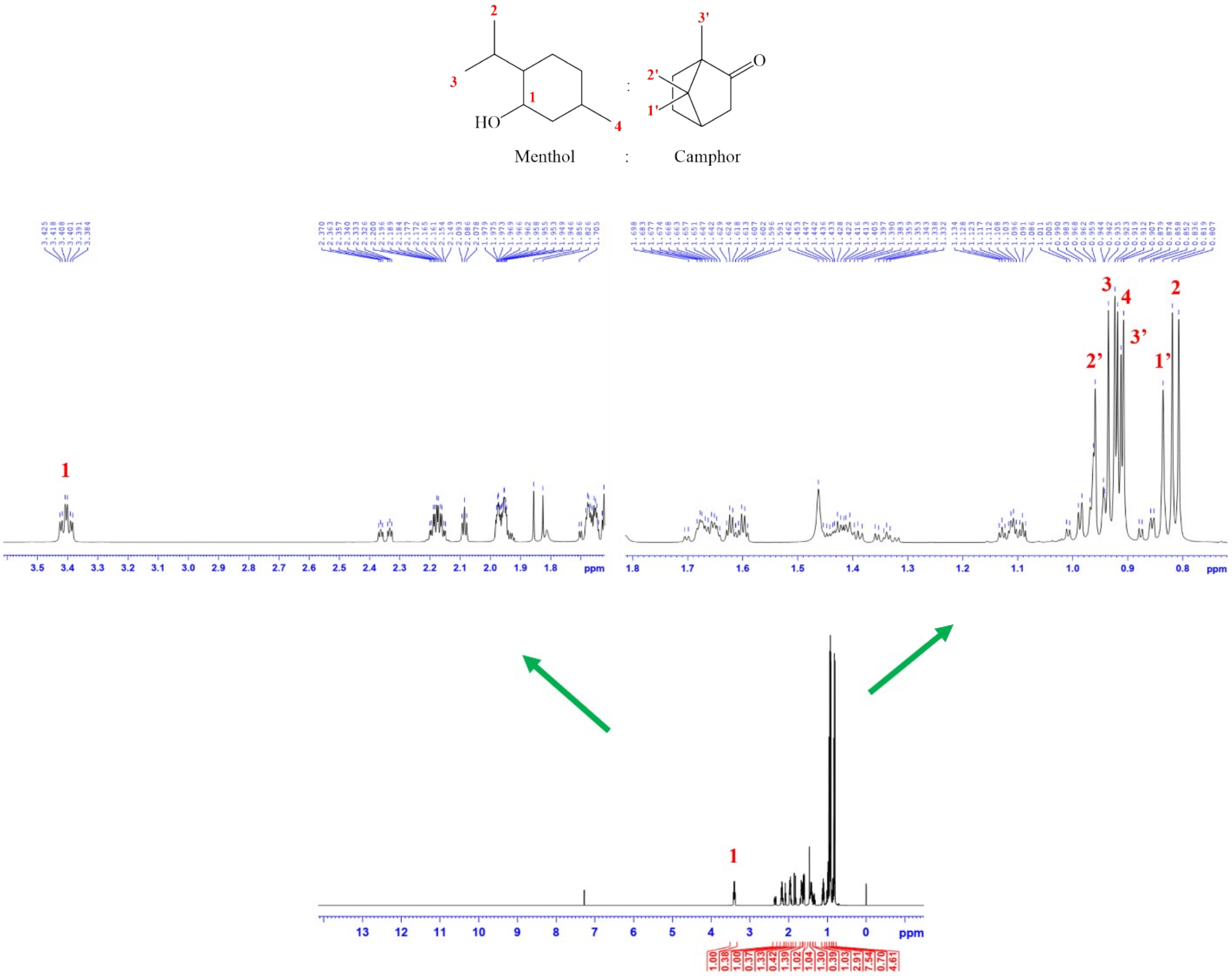

Figure S11. ^{1}H NMR spectra of recycled Men-CP.

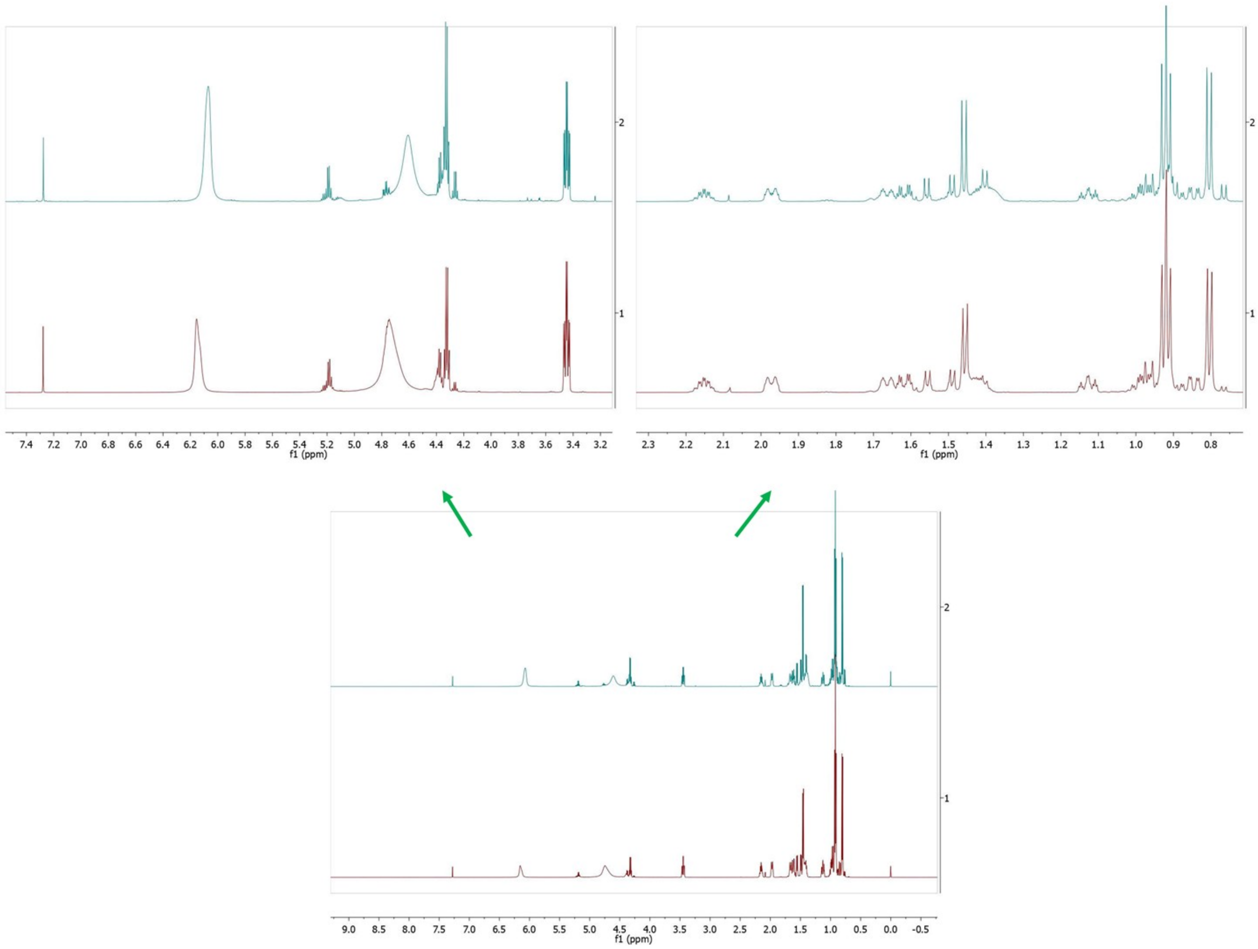

Figure S12. Stacked ^{1}H NMR spectra of fresh (red) and recycled (green) Men–LA HDESs, including full-spectrum and expanded views for direct comparison of spectral profiles.

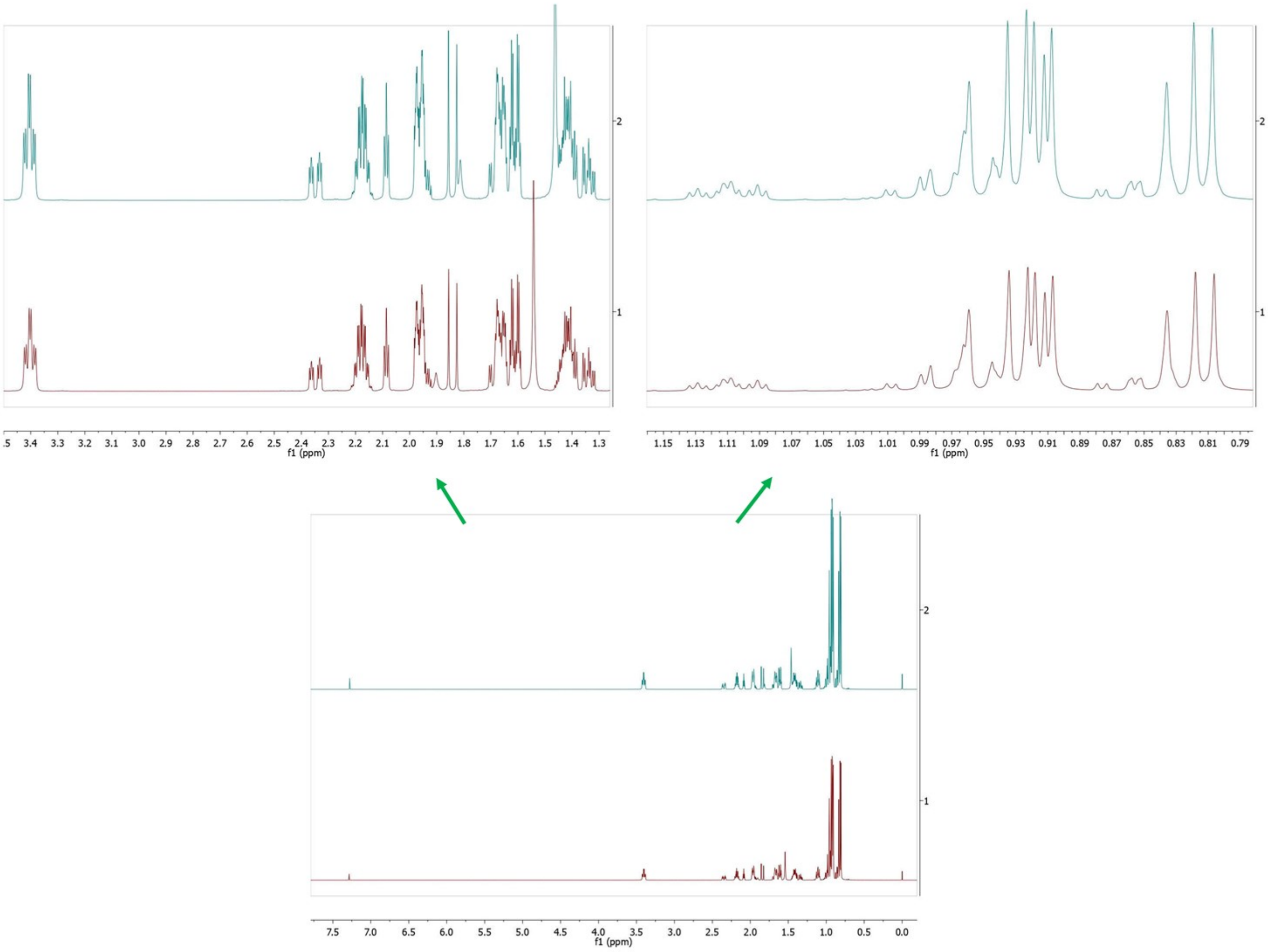

Figure S13. Stacked ^{1}H NMR spectra of fresh (red) and recycled (green) Men–CP HDESs, including full-spectrum and expanded views for direct comparison of spectral profiles.
